# Approach to an Initial Oncologic Patient Encounter: A Simulation-Based Training for First-Year Medical Students

**DOI:** 10.15766/mep_2374-8265.11574

**Published:** 2026-04-24

**Authors:** Allan John R. Barcena, Marvin R. Bernardino, Dominic Karl M. Bolinas, Kitz Paul D. Marco, Shelita Kimble, James Cavalier, Michael K. Rooney, Marites P. Melancon, Jillian R. Gunther

**Affiliations:** 1 Postdoctoral Fellow, Department of Interventional Radiology, The University of Texas MD Anderson Cancer Center; 2 Research Data Coordinator, Department of Interventional Radiology, The University of Texas MD Anderson Cancer Center; 3 Graduate Student, Department of Interventional Radiology, The University of Texas MD Anderson Cancer Center; 4 Director, Simulation Center, Teaching, Inter-Professional, and Simulation (TIPS) Education Center, The University of Texas MD Anderson Cancer Center; 5 Associate Vice President, Education Operations, Teaching, Inter-Professional, and Simulation (TIPS) Education Center, The University of Texas MD Anderson Cancer Center; 6 Assistant Professor, Department of Radiation Oncology, The University of Texas MD Anderson Cancer Center; 7 Professor, Department of Interventional Radiology, The University of Texas MD Anderson Cancer Center; 8 Associate Professor, Department of Radiation Oncology, The University of Texas MD Anderson Cancer Center

**Keywords:** Simulation, Cancer Diagnosis, Communication Skills, History-Taking, Oncology

## Abstract

**Introduction:**

During an initial oncologic encounter, care providers must convey information and plans with confidence and empathy. First-year medical students often lack opportunities to develop the skills necessary for these challenging encounters. We developed a novel simulation-based training designed to prepare early-level medical students to demonstrate empathy, communicate care plans, and deliver bad news during initial oncologic patient visits.

**Methods:**

Medical students in a summer research program participated. The session included a didactic, followed by 2 simulated patient encounters: a young woman with lymphoma and an older man with rectal cancer. Students rotated through roles as primary provider, secondary provider, family member, and observer. Each simulation was followed by a faculty-led debriefing. We collected postsession feedback using a mixed-methods evaluation. The activity lasted approximately 2 hours.

**Results:**

Of the 54 participants, 49 (91%) completed the evaluation. The results were highly positive, with 100% of respondents agreeing or strongly agreeing that the simulation helped them apply knowledge practically and that they would use these skills in future practice. Qualitative feedback highlighted improvements in communication skills, the importance of empathy, and the need for adaptability. Participants identified the debriefing sessions, standardized patients, and active participation as the most valuable components.

**Discussion:**

This simulation is effective for preparing medical students for the complexities of initial oncologic patient encounters. It provides a safe environment for the practice and refinement of essential clinical and interpersonal skills, fostering a more empathetic and competent approach to patient care.

## Educational Objectives

By the end of this activity, learners will be able to:

1. Conduct a comprehensive history for a patient with a new cancer diagnosis, incorporating clinical, social, and emotional aspects of care.
2. Demonstrate empathetic communication skills when delivering a cancer diagnosis and discussing initial treatment considerations.
3. Collaborate effectively in a team-based clinical setting to address patient and family member concerns during a sensitive encounter.
4. Reflect on their performance and identify areas for improvement in patient communication and clinical reasoning through a structured debriefing session.
5. Recognize the distinct psychosocial needs of patients with different cancer diagnoses and demographic backgrounds.

## Introduction

The initial diagnosis of cancer can be one of the most challenging moments in a patient's life, and how health care providers communicate this diagnosis plays a critical role in shaping the patient's psychological responses, decision-making process, and overall experience. In recognition of this, medical education has increasingly emphasized the importance of communication skills, particularly in oncology, where the delivery of a cancer diagnosis and discussion of initial management options carry profound emotional and psychological consequences for patients.^1–4^

Despite this emphasis, medical students, especially those early in training, are often unprepared for the emotional weight of delivering sensitive news, such as a cancer diagnosis.^5^ Although students develop a solid foundation in clinical knowledge during their first year, opportunities to practice complex interpersonal skills, including history-taking and delivering bad news, remain limited in traditional curricula.^5^ Recent literature suggests that simulation-based communication skills can help students better navigate emotionally charged clinical encounters more effectively and improve patient outcomes.^6,7^

Simulation-based education is a well-established and highly effective tool in medical training, providing learners with a safe, controlled environment to develop both clinical and communication competencies.^6–9^ Simulation has been shown to improve student performance in patient communication, a skill that is particularly critical in oncology, where the patient's emotional state and understanding of their diagnosis can significantly influence treatment decisions.^10^ Building on prior simulation-based training in breaking bad news,^11,12^ we designed a simulation-based activity that shifts the focus to the initial oncologic intake, which requires integrating history-taking, delivering a new cancer diagnosis, and forward planning.

In this article, we describe a novel simulation activity for first-year medical students, focusing on an initial history-taking encounter with a patient recently diagnosed with cancer. Unlike existing curricula that focus primarily on breaking bad news for residents or advanced learners, our simulation targets first-year medical students and emphasizes the comprehensive nature of the initial intake. The activity intentionally integrates clinical history-taking with communication skills training, allowing students to practice navigating one of the most challenging yet essential aspects of oncology care. The goal is to prepare students for future patient interactions, ensuring they approach such sensitive encounters with empathy, professionalism, and a comprehensive understanding of the emotional impact of a cancer diagnosis.

## Methods

### Activity Design and Development

Academic faculty with a focus on and appointments in medical student education at a comprehensive cancer center collaborated with simulation specialists to design this activity. An informal survey with medical students from 3 different institutions was conducted to better understand experiences with oncology-focused education and simulation during the typical medical school curriculum. The existing published literature including *MedEdPORTAL* resources was reviewed to understand available educational tools similar to the intended topics. Resources with partially similar intentions (e.g., oncologic history-taking, breaking bad news) were reviewed, and portions were used, as appropriate. The activity was grounded in Kolb's experiential learning cycle, guiding learners through concrete experience (the simulation), reflective observation (the debrief), and abstract conceptualization (didactics), thereby preparing them for active experimentation in future clinical roles.^13^

### Curricular Context and Learner Preparation

We invited first-year medical students participating in an oncology-focused summer research program at the University of Texas MD Anderson Cancer Center to participate in the simulation-based training. Per an informal survey of students, training in simulated patient encounters, communication skills, and breaking bad news varied; the majority experienced prior simulated encounters unrelated to oncology. Faculty facilitators were clinicians with at least 2 years of oncology and/or medical education experience. We conducted simulations during the summer of 2024 and 2025. The MD Anderson Cancer Center Institutional Review Board declared this educational activity exempt.

### Presimulation Didactic

All students first attended a 30-minute in-person didactic session that was adapted from a SlideShare presentation by McMillan^14^ (Appendix A) to teach them how to perform an initial oncologic patient encounter, including taking an oncologic history. Additionally, the session covered other essential components of patient interviews, including social, financial, and mental/emotional aspects of care. Briefly, we presented the major categories of oncologic treatments (eg, systemic therapy, radiation therapy), along with a general overview of logistics and potential side effects. Specific attention was given to recognizing the psychological and emotional responses patients may experience upon learning of a cancer diagnosis. The didactic aimed to equip students with the knowledge and communication skills necessary for a sensitive and thorough oncology history. We provided students with interview guides (Appendix B) for reference during the sessions. During 2025, we provided this guide at the presimulation didactic to allow for ample review time.

### Simulation Design

We identified standardized patients from staff members of the MD Anderson Cancer Center Simulation Center (Year 1) or from members of a Patient and Family Advisory Program (Year 2). Once selected, they underwent structured training led by medical educators to ensure they could accurately and reliably simulate medical conditions, emotional states, and patient histories. Training lasted approximately 2 hours and included instruction on case scripts, role-play exercises, feedback techniques, and assessment calibration to ensure consistency across encounters. This preparation allowed the standardized patients to provide realistic, repeatable experiences for medical learners in clinical teaching and assessment settings.

### Implementation

**Figure 1.**
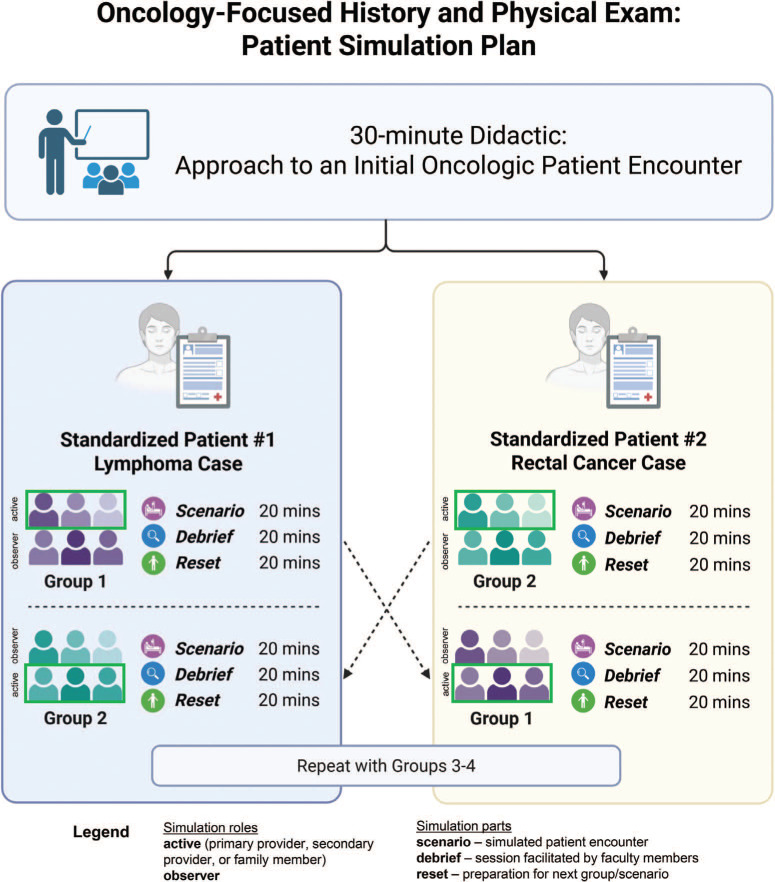
Schematic diagram for the simulation experience design and plan strategy.

We conducted the simulation in 2 parts; each part involved the students meeting a patient with a new cancer diagnosis. After the predidactic simulation, we randomly assigned the students to the simulation rooms (Figure 1) and oriented them to the room setup and planned interaction. Standardized patients used a written script^11,12^ (Appendix C) containing the chief complaint, patient medical history, vitals, and initial workup results. Each student participated in 1 encounter as the primary provider, secondary provider, family member, or observer. If not assigned a participant role, students observed from the control room. The facilitators observed while the students were conducting their interviews. The ratio of learners to faculty facilitators was approximately 6:1. Due to time constraints, students did not rotate through all 4 roles; instead, they participated in 1 active role (provider or family) and 1 observer role, or rotated roles between case 1 and case 2 to ensure that every student had an active role in at least 1 scenario. Given the learners’ early level, the focus on communication, and time constraints, physical examinations were not performed. We provided findings such as imaging or endoscopy results.

We designed the 2 cases to expose students to different cancer diagnoses and varied patient demographics, highlighting distinct communication needs and patient concerns. Each student completed both simulations during a single 2-hour session.

### Case 1: Lymphoma Diagnosis (Young Woman)

The first case involved an encounter with a young woman in her late 20s who had just been diagnosed with lymphoma. The scenario emphasized the emotional impact of the diagnosis on a young patient, addressing concerns about fertility, treatment options, and long-term prognosis. The students acting as primary providers were expected to deliver the complex diagnosis while responding to the patient's emotional reactions, addressing her questions with empathy and clarity.

### Case 2: Rectal Cancer Diagnosis (Older Man)

The second case featured an older man in his late 60s who was recently diagnosed with rectal cancer. This case emphasized the importance of addressing both clinical aspects of the diagnosis and social considerations such as family support, potential financial burdens, and age-related concerns about treatment. Students in the primary provider role were tasked with balancing the technical details of treatment options with empathetic communication about the patient's fears and future quality of life.

### Simulation Roles

During the simulation, we assigned students to 1 of the following roles with specific instructions provided to ensure engagement:

- Primary provider: Responsible for leading the encounter, taking the patient's history, and delivering the cancer diagnosis (aligns with Objectives 1 and 2).
- Secondary provider: Assisted the primary provider and observed the interaction, offering support, scribing key details, or looking up additional information as needed (aligns with Objective 3).
- Family member: Played the role of a relative (eg, spouse, parent) present during the consultation, adding emotional and social dynamics to the encounter (aligns with Objective 5).
- Observer: Watched the simulation without direct involvement. Observers were asked to note communication behaviors and offer feedback during the debrief session.

Each role allowed students to experience the scenario from different perspectives, enhancing their understanding of the emotional and interpersonal aspects of the patient-provider-family interaction.

### Debrief and Reflection

Following the conclusion of each case, we held a debrief session (aligns with Objective 4) to allow students to reflect on their experiences using a debrief guide (Appendix D). Faculty facilitators led the debrief, focusing on the communication strategies used during the encounter, the challenges encountered, and the emotional dynamics of delivering a cancer diagnosis. Learner assessment was formative, occurring in real time during the debrief, when faculty provided specific feedback on performance against the learning objectives. Facilitators used the “debriefing with good judgment” approach, which combines genuine inquiry with rigorous reflective feedback.^15^ We encouraged students to share their feelings and insights from their roles and discuss how they would approach similar situations in the future.

### Data Collection

We collected postsession anonymous feedback from students through written evaluations (Appendix E and Appendix F) containing quantitative and qualitative items designed to assess the overall learning experience and effectiveness of the simulation. Quantitative feedback was gathered using 5-point Likert scale questions (1 = *strongly agree* and 5 = *strongly disagree*). Statistical analysis was performed using GraphPad Prism 10.3.1, and descriptive statistics including median and interquartile range were reported. Mann-Whitney test compared subgroups. Statistical significance was defined as *P* < .05. Qualitative feedback was collected via open-ended questions, with results first reviewed independently by Allan John Barcena and Jillian Gunther to identify common themes; these were then refined during team discussion.

## Results

**Figure 2.**
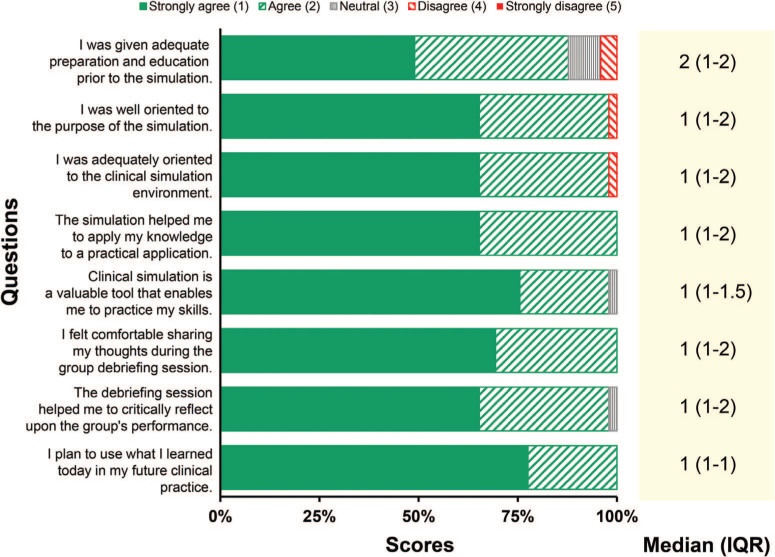
Summary of responses regarding the simulation experience from the medical students (*N* = 49).

Fifty-four students completed the presimulation didactic, of which 49 (91%) responded to the postsimulation evaluation. The students broadly rated the experience positively, with detailed response characteristics to the 5-point Likert scale questions summarized in Table 1 and Figure 2. There were no significant differences in responses between participants and observers (all *P* > .05). When comparing the second year to the first year of the simulation, students reported higher levels of agreement with having adequate preparation for the session (median 1 vs 2, *P* = .599).

**Table 1.**
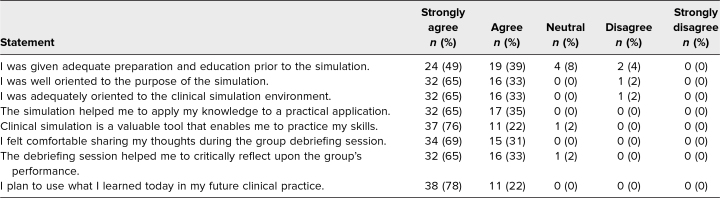
Quantitative Feedback on the Simulation Experience via Likert Scale Questions (*N* = 49)

Based on the qualitative feedback (Table 2), students frequently reported learning communication skills such as listening, addressing patient concerns, and speaking in layman's terms (Objectives 1, 2, and 5). One student noted the importance of “silence” and allowing the patient to process information before moving on. Others emphasized the value of avoiding medical jargon and “speaking plainly” to ensure patient understanding. Participants also highlighted the importance of empathy and building rapport through actions such as showing compassion, using nonverbal cues, and maintaining eye contact (Objectives 1 and 2). One participant reflected that “matching the patient's emotional energy” is critical, whereas another noted that “getting close to the patient and keeping eye contact shows you're paying attention and you care.” The third major takeaway was the need for flexibility and adaptability during patient encounters to meet patients’ needs (Objectives 1 and 5). Learners described realizing the need to “be nimble with how you approach the conversation” and focusing on “connecting with the patient rather than following a script.”

**Table 2.**
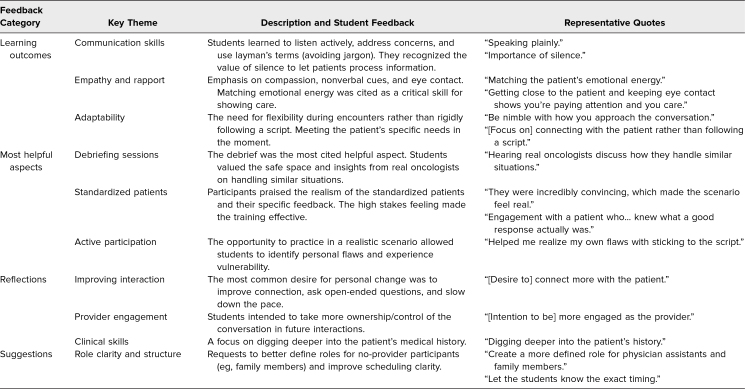
Qualitative Feedback on the Simulation Experience

Participants described the simulation's most helpful aspect as the debriefing and feedback sessions that occurred after the encounters (Objective 4). Most participants affirmed that the facilitators helped create a safe space for discussion. Students specifically valued “hearing real oncologists discuss how they handle similar situations,” noting that the debrief provided “critical feedback for all participants.” The second most valued component was the standardized patients, who were commended for their realistic portrayals and insightful feedback (Objective 5). “They were incredibly convincing, which made the scenario feel real and the stakes feel high,” wrote 1 student. Another appreciated “the engagement with a patient who actually went through the process and knew what a good response actually was.” The opportunity for active participation in a realistic scenario was also highly valued (Objectives 1, 2, and 3); one learner commented, “This was a really great simulation that helped me realize my own flaws with ‘sticking to the script’ and reminded me about the vulnerability of my patient encounters.”

When reflecting on what they would change as a participant, they most commonly desired to improve communication and patient interaction. This included goals like connecting more with the patient, asking more open-ended questions, and slowing down. The next most frequent theme involved taking more control of the conversation and being more engaged as the provider. The third theme focused on improving clinical skills, such as delving deeper into the patient's history. The most frequent suggestion for improvement was to enhance role clarity and the overall structure, with comments on better defining roles for nonprovider participants and on ensuring that family members were more involved. Specific comments included a desire to “create a more defined role for physician assistants and family members” and requests to “let the students know the exact timing of simulation” to facilitate better scheduling.

## Discussion

We developed and evaluated a novel simulation activity designed to teach first-year medical students how to conduct an initial history-taking encounter for patients newly diagnosed with cancer. The results of our evaluation demonstrated that the students responded positively to the simulation, with increased confidence and a better understanding of both the clinical and emotional components of delivering a cancer diagnosis. The feedback also indicated that the simulation was effective in preparing students for real-world clinical encounters.

Simulation-based learning is an increasingly important tool in medical education, particularly for teaching communication skills necessary in complex, emotionally challenging situations such as delivering a cancer diagnosis.^16^ Proper presimulation education and preparation can enhance the experience. The majority of students reported feeling well prepared for the session with the given materials and guidance. In response to some students’ unease (having not yet completed clinical training), we provided the interview guides earlier in the day during the second year, which likely contributed to the greater feelings of preparedness reflected in the surveys. To further strengthen preparation in future iterations, we plan to incorporate photographs from prior simulations to help students visualize the encounter and provide a video resource as an example of breaking bad news, which is one of the more challenging aspects of the simulation.^17^ Furthermore, most students indicated that the orientation clarified the exercise's goals, enabling them to approach the simulation with a clear understanding of expectations. This again highlights the importance of thorough preparation and orientation in maximizing the educational value of simulation-based exercises.

Simulation provides students with the opportunity to apply theoretical knowledge in a practical setting. In response to the question regarding knowledge transfer to actual practice, students reported that they felt the simulation effectively bridged the gap between classroom learning and real-world clinical medicine. The 2 distinct cancer cases—lymphoma in a young woman and rectal cancer in an older man—challenged students to synthesize clinical data while managing the flow of a complex interview. The simulation also challenged the students to consider both the medical and psychosocial aspects of patient care, reinforcing the importance of empathy and communication in oncology.

The role-playing aspect of the simulation was also a crucial component of the learning experience. By rotating through different roles (primary provider, secondary provider, family member, observer), students experienced the consultation from multiple perspectives. This experience helped them appreciate the complexity of patient-provider-family dynamics in cancer care. Consistent with previous literature, the positive feedback suggests that role-playing in simulation enhances communication skills, fosters empathy, and prepares students for real-world clinical encounters.^18–20^ These findings were strongly supported by participants, who identified improved communication and empathy as the top 2 concepts learned during the simulation.

The debriefing session following each simulation encounter was another key aspect of the exercise. Most students reported feeling comfortable expressing their reflections and engaging in group discussions during the debriefing session. This is an important finding, as a supportive environment for open reflection is essential for effective learning.^21^ The debriefing session successfully allowed students to critically reflect upon their performance. Debriefing has been shown to enhance the learning experience by promoting self-awareness and identifying areas for improvement.^6^ The structured yet open-ended nature of the debriefing facilitated constructive feedback and allowed students to learn from their own experiences and those of their peers. This collaborative reflection is crucial in refining communication strategies, particularly in oncology, where understanding the emotional needs of patients is paramount.

One of the most promising outcomes of this activity was the students’ expressed intention to incorporate the lessons learned to their future clinical practice. The simulation supported the development of clinical skills while deepening their appreciation for the importance of empathetic communication in oncology care. This finding aligns with previous work showing that simulation-based experiences can yield long-term benefits for both clinical skills and professional attitudes.^6^

While these results are encouraging, there are areas for future improvement. First, the activity was implemented with a relatively small single-institution cohort of students, so adapting it for other institutions may require modification. Generalizability may also be limited by the fact that our participants were enrolled in an oncology-focused summer program and may have higher baseline motivation than the general medical student population. Additionally, the evaluation relied on self-reported feedback, which may be subject to bias. Future iterations could incorporate objective performance measures, such as direct assessment by standardized patients and/or facilitators, to gain a more comprehensive understanding of the simulation's impact on communication and clinical skills. A presession survey could also assess student confidence and prior experience with this type of activity and allow for pre/postsession comparison. In future sessions, the survey questions will be slightly modified to reduce bias.

Future work should also explore the longitudinal impact of this simulation. Longitudinal follow-up could assess whether the lessons learned during the simulation translate into more effective communication with real patients and whether students who participate in simulations are better prepared to handle emotionally difficult clinical encounters.

### Conclusion

This educational innovation demonstrates that simulation-based education is a valuable tool for teaching early-level medical students how to handle complex patient interactions, particularly in oncology. The positive student feedback regarding the application of knowledge, role-playing, and debriefing sessions suggests that simulation is an effective method for enhancing both clinical and communication skills. By integrating these simulations into early medical education, we can better prepare students to navigate the emotional and psychosocial challenges of oncology care, ultimately improving patient-provider interactions and patient outcomes.

## Appendices


Approach to an Initial Oncologic Patient Encounter.pptxCase Guide for Students.docxCase Information.docxDebrief Guide for Sim Facilitator.docxPostsimulation Evaluation (Original).docxPostsimulation Evaluation (Revised).docx
*All appendices are peer reviewed as integral parts of the Original Publication.*
