# Supplementary material for: Approach to an Initial Oncologic Patient Encounter: A Simulation-Based Training for First-Year Medical Students
Source: MedEdPORTAL. 2026 Apr 24;22:11574. doi: 10.15766/mep_2374-8265.11574 (PMC13106612; doi:10.15766/mep_2374-8265.11574)
Supplement: Supplementary file 1 — Approach to an Initial Oncologic Patient Encounter.pptxCase Guide for Students.docxCase Information.docxDebrief Guide for Sim Facilitator.docxPostsimulation Evaluation (Original).docxPostsimulation Evaluation (Revised).docx [file mep_2374-8265.11574-s001.zip › A. Approach to an Initial Oncologic Patient Encounter.pptx]

## Slide 1
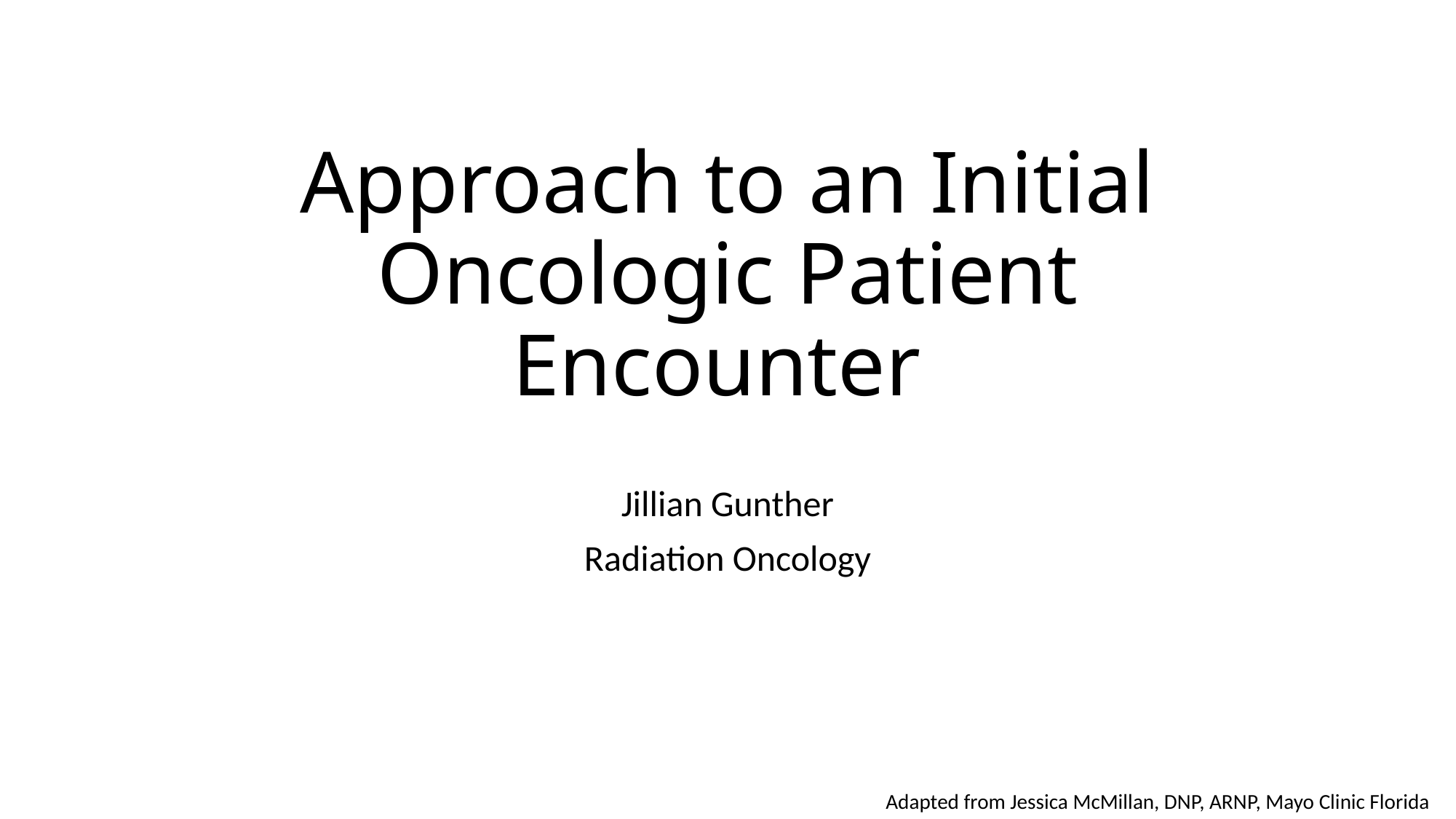

# Approach to an Initial Oncologic Patient Encounter
Jillian Gunther
Radiation Oncology
Adapted from Jessica McMillan, DNP, ARNP, Mayo Clinic Florida

## Slide 2
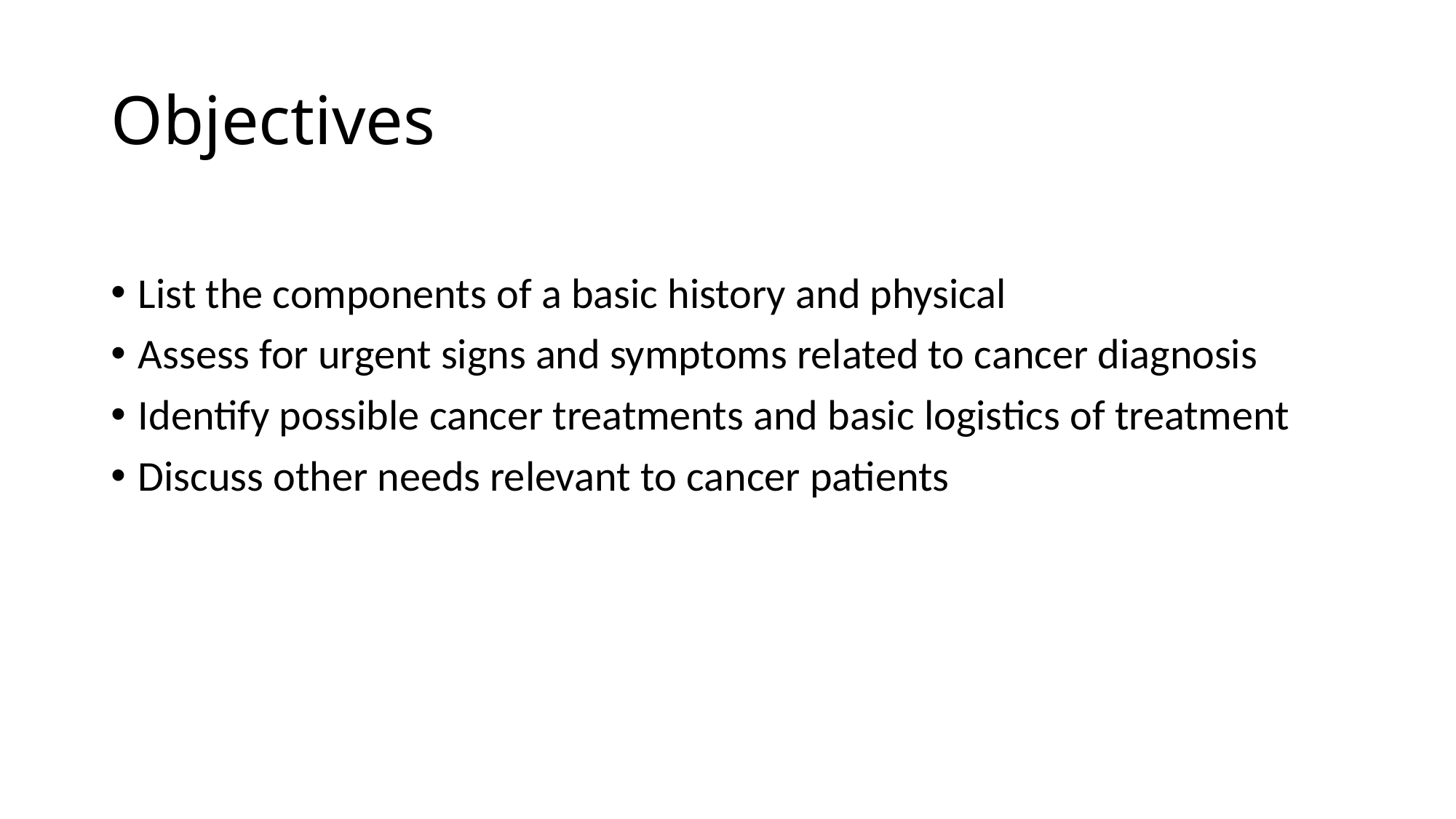

# Objectives
List the components of a basic history and physical
Assess for urgent signs and symptoms related to cancer diagnosis
Identify possible cancer treatments and basic logistics of treatment
Discuss other needs relevant to cancer patients

## Slide 3
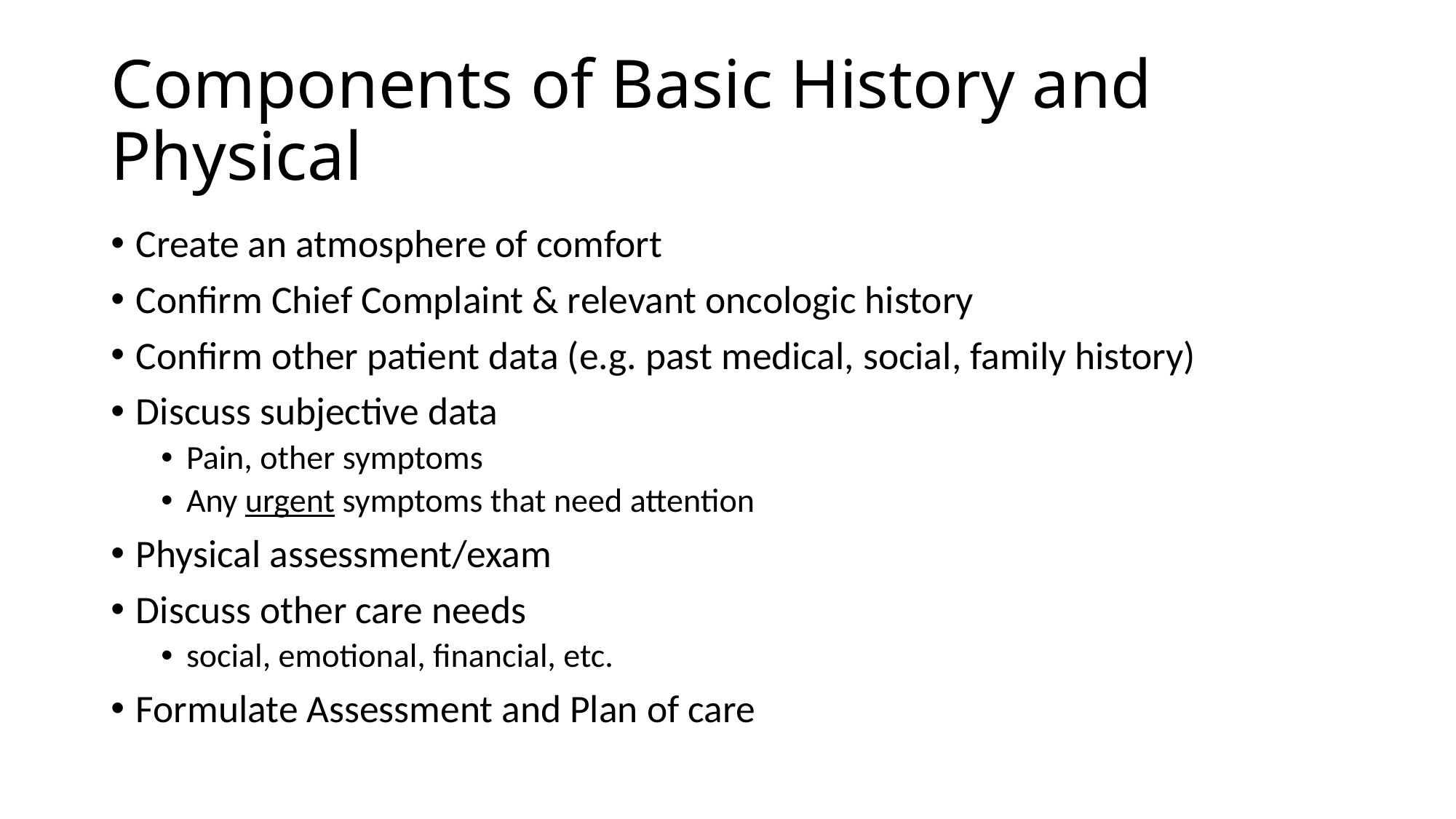

# Components of Basic History and Physical
Create an atmosphere of comfort
Confirm Chief Complaint & relevant oncologic history
Confirm other patient data (e.g. past medical, social, family history)
Discuss subjective data
Pain, other symptoms
Any urgent symptoms that need attention
Physical assessment/exam
Discuss other care needs
social, emotional, financial, etc.
Formulate Assessment and Plan of care

## Slide 4
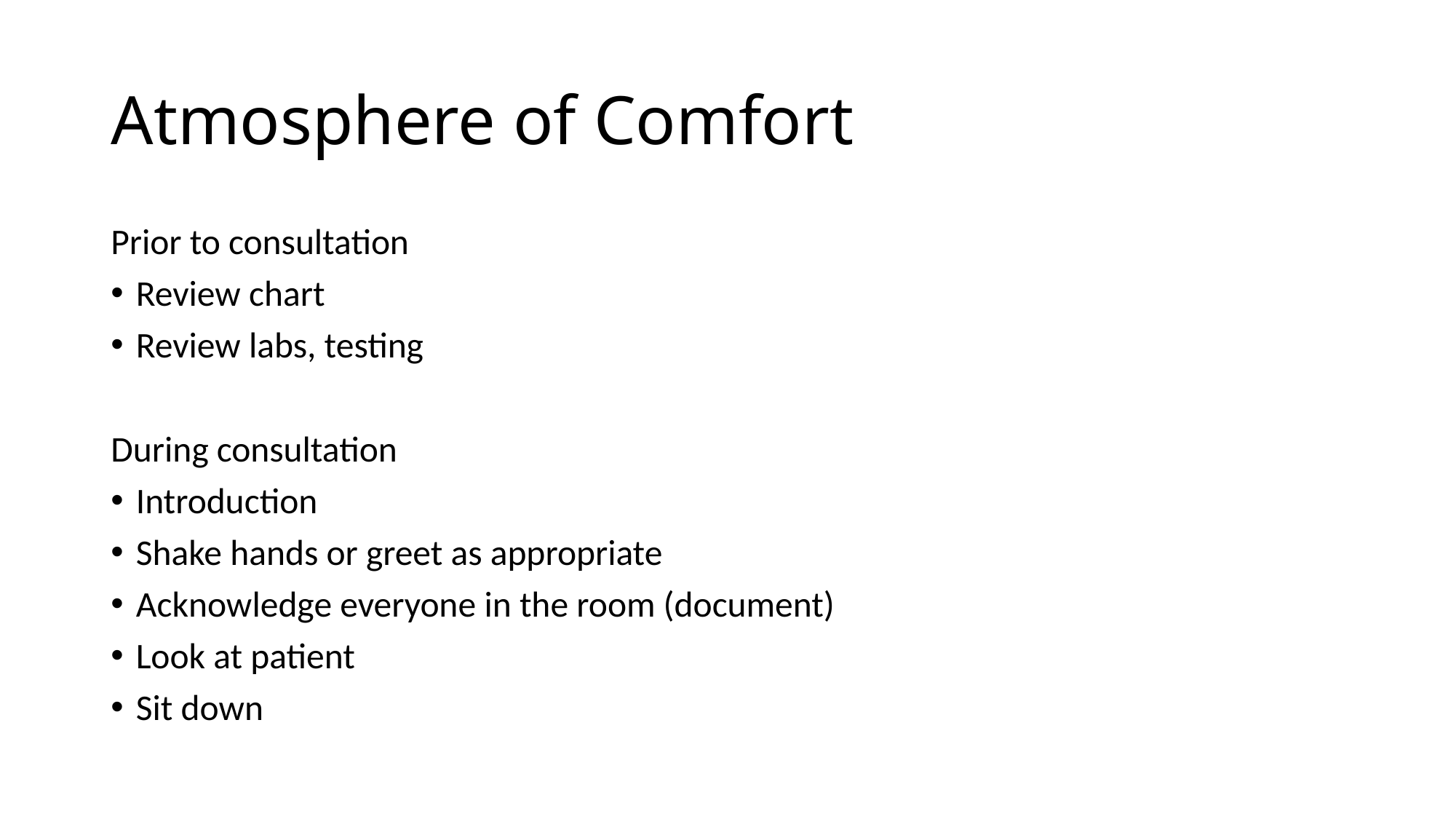

# Atmosphere of Comfort
Prior to consultation
Review chart
Review labs, testing
During consultation
Introduction
Shake hands or greet as appropriate
Acknowledge everyone in the room (document)
Look at patient
Sit down

## Slide 5
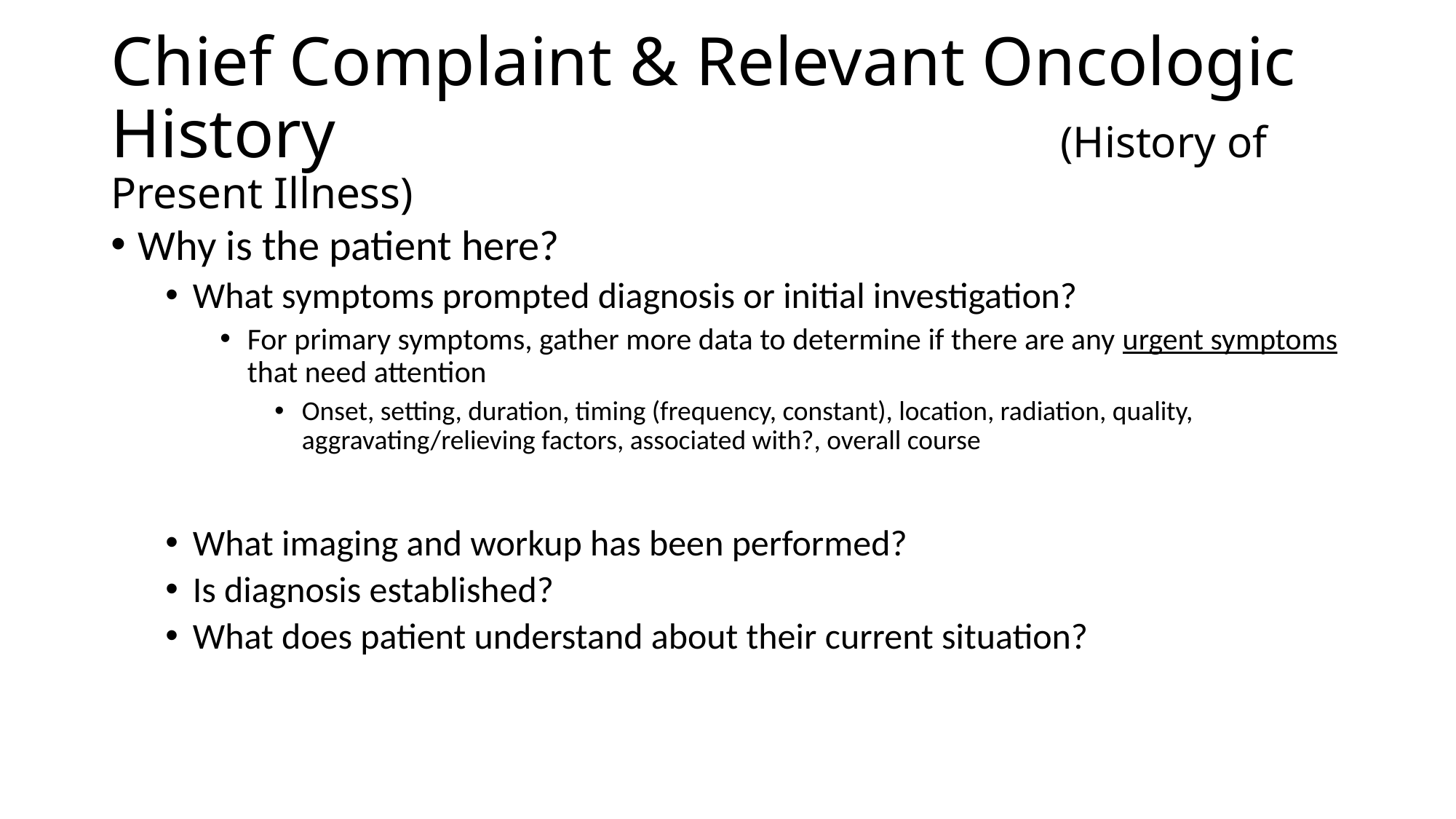

# Chief Complaint & Relevant Oncologic History 	 (History of Present Illness)
Why is the patient here?
What symptoms prompted diagnosis or initial investigation?
For primary symptoms, gather more data to determine if there are any urgent symptoms that need attention
Onset, setting, duration, timing (frequency, constant), location, radiation, quality, aggravating/relieving factors, associated with?, overall course
What imaging and workup has been performed?
Is diagnosis established?
What does patient understand about their current situation?

## Slide 6
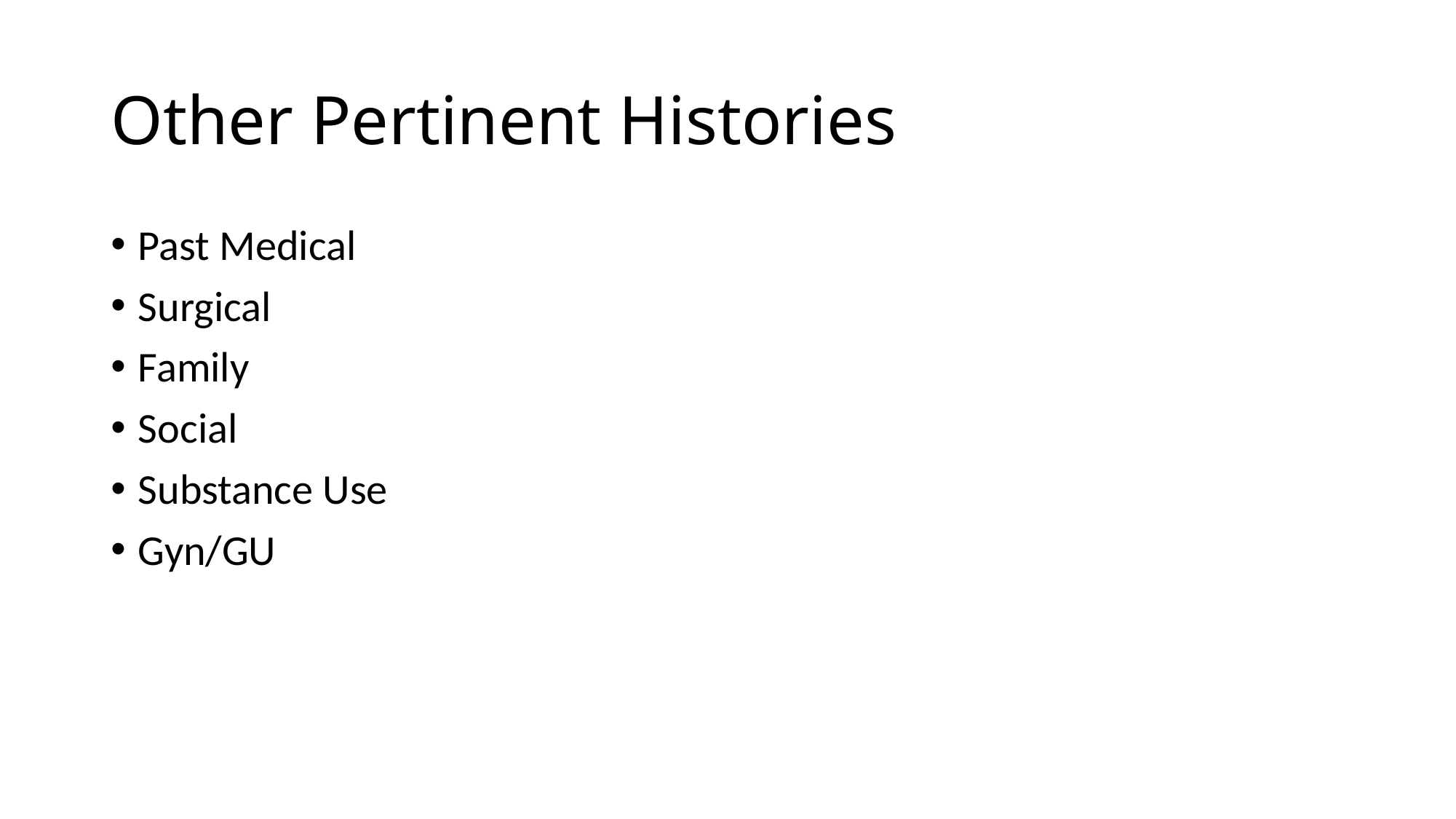

# Other Pertinent Histories
Past Medical
Surgical
Family
Social
Substance Use
Gyn/GU

## Slide 7
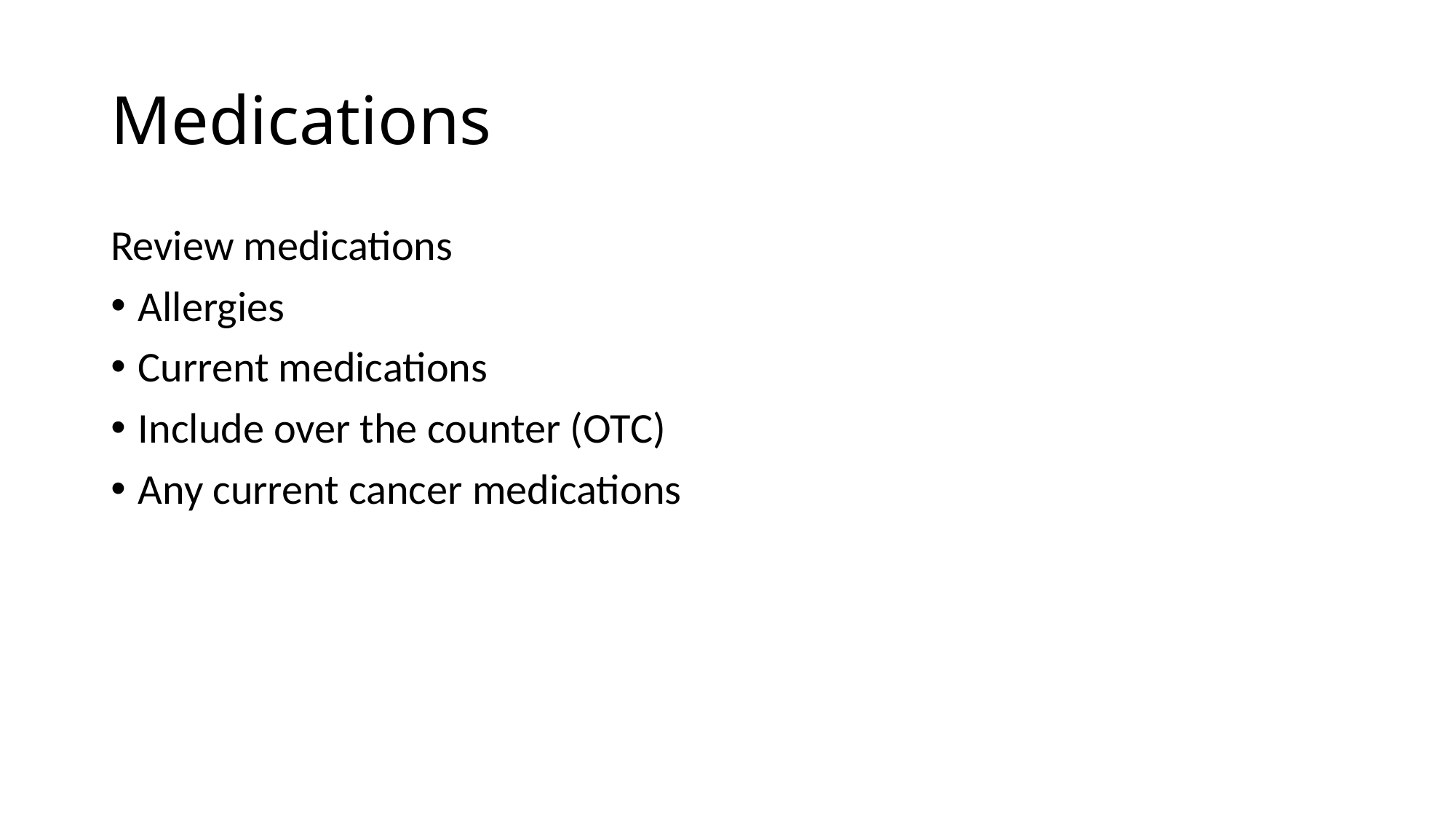

# Medications
Review medications
Allergies
Current medications
Include over the counter (OTC)
Any current cancer medications

## Slide 8
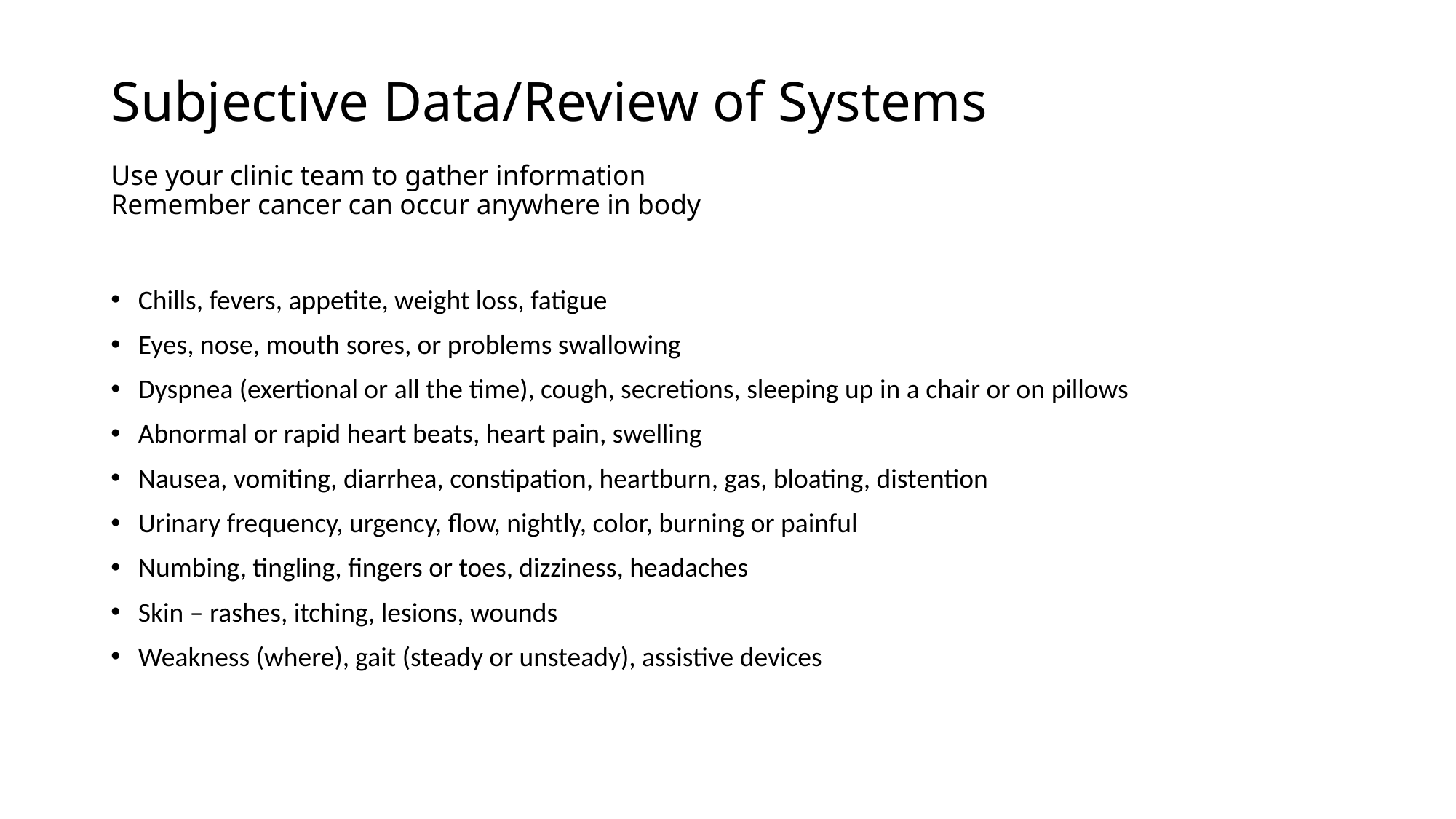

# Subjective Data/Review of Systems Use your clinic team to gather information Remember cancer can occur anywhere in body
Chills, fevers, appetite, weight loss, fatigue
Eyes, nose, mouth sores, or problems swallowing
Dyspnea (exertional or all the time), cough, secretions, sleeping up in a chair or on pillows
Abnormal or rapid heart beats, heart pain, swelling
Nausea, vomiting, diarrhea, constipation, heartburn, gas, bloating, distention
Urinary frequency, urgency, flow, nightly, color, burning or painful
Numbing, tingling, fingers or toes, dizziness, headaches
Skin – rashes, itching, lesions, wounds
Weakness (where), gait (steady or unsteady), assistive devices

## Slide 9
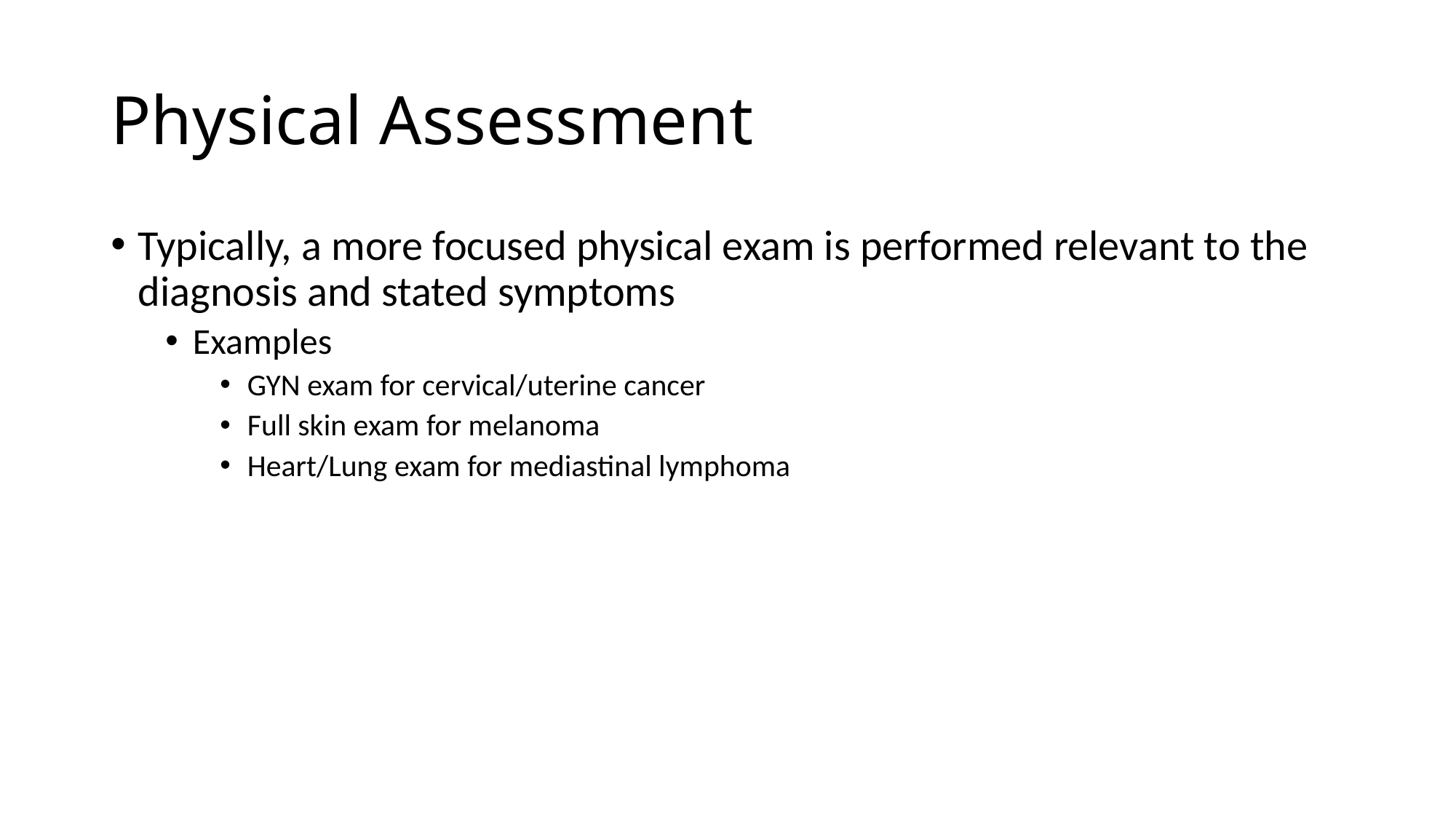

# Physical Assessment
Typically, a more focused physical exam is performed relevant to the diagnosis and stated symptoms
Examples
GYN exam for cervical/uterine cancer
Full skin exam for melanoma
Heart/Lung exam for mediastinal lymphoma

## Slide 10
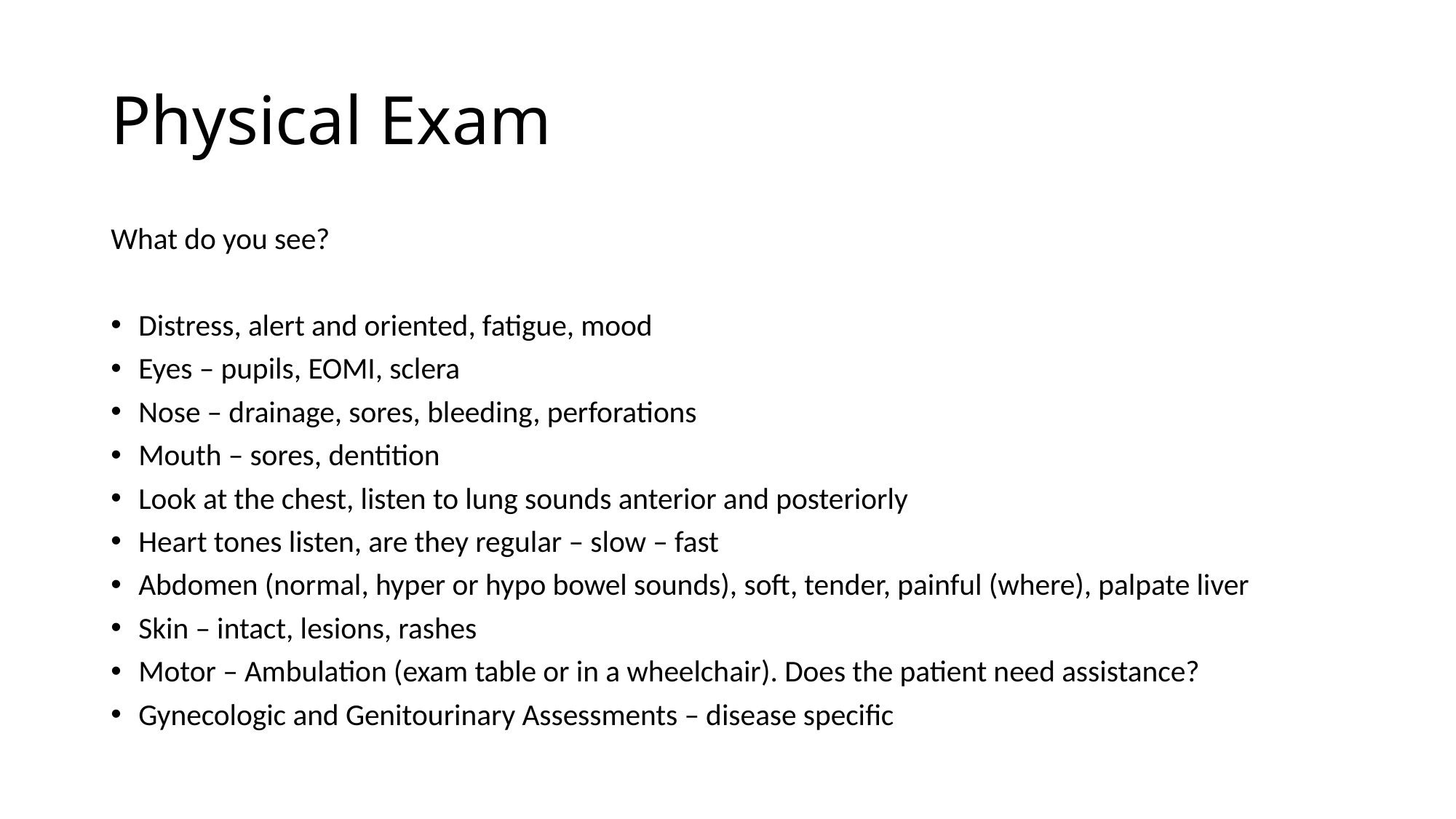

# Physical Exam
What do you see?
Distress, alert and oriented, fatigue, mood
Eyes – pupils, EOMI, sclera
Nose – drainage, sores, bleeding, perforations
Mouth – sores, dentition
Look at the chest, listen to lung sounds anterior and posteriorly
Heart tones listen, are they regular – slow – fast
Abdomen (normal, hyper or hypo bowel sounds), soft, tender, painful (where), palpate liver
Skin – intact, lesions, rashes
Motor – Ambulation (exam table or in a wheelchair). Does the patient need assistance?
Gynecologic and Genitourinary Assessments – disease specific

## Slide 11
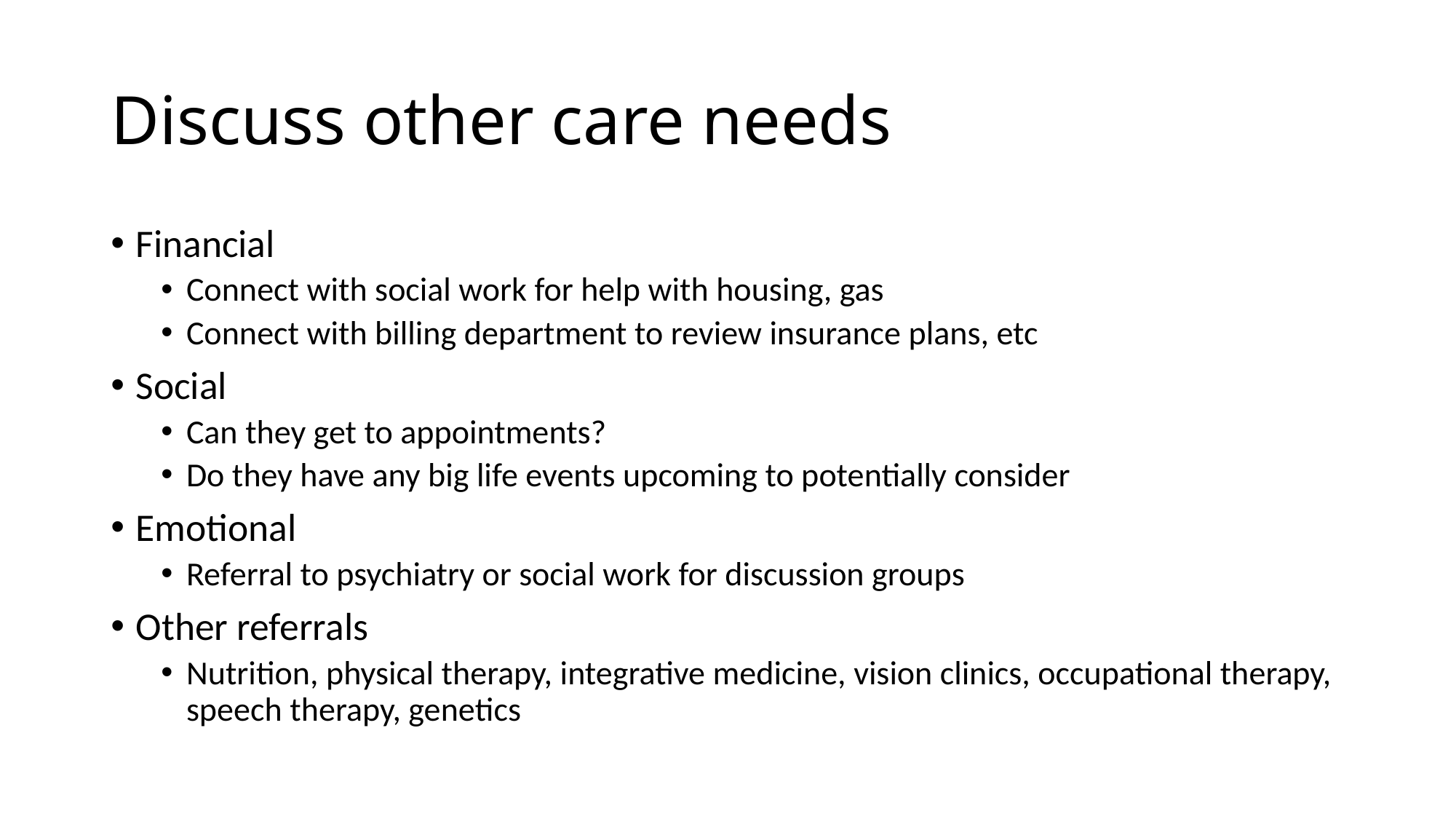

# Discuss other care needs
Financial
Connect with social work for help with housing, gas
Connect with billing department to review insurance plans, etc
Social
Can they get to appointments?
Do they have any big life events upcoming to potentially consider
Emotional
Referral to psychiatry or social work for discussion groups
Other referrals
Nutrition, physical therapy, integrative medicine, vision clinics, occupational therapy, speech therapy, genetics

## Slide 12
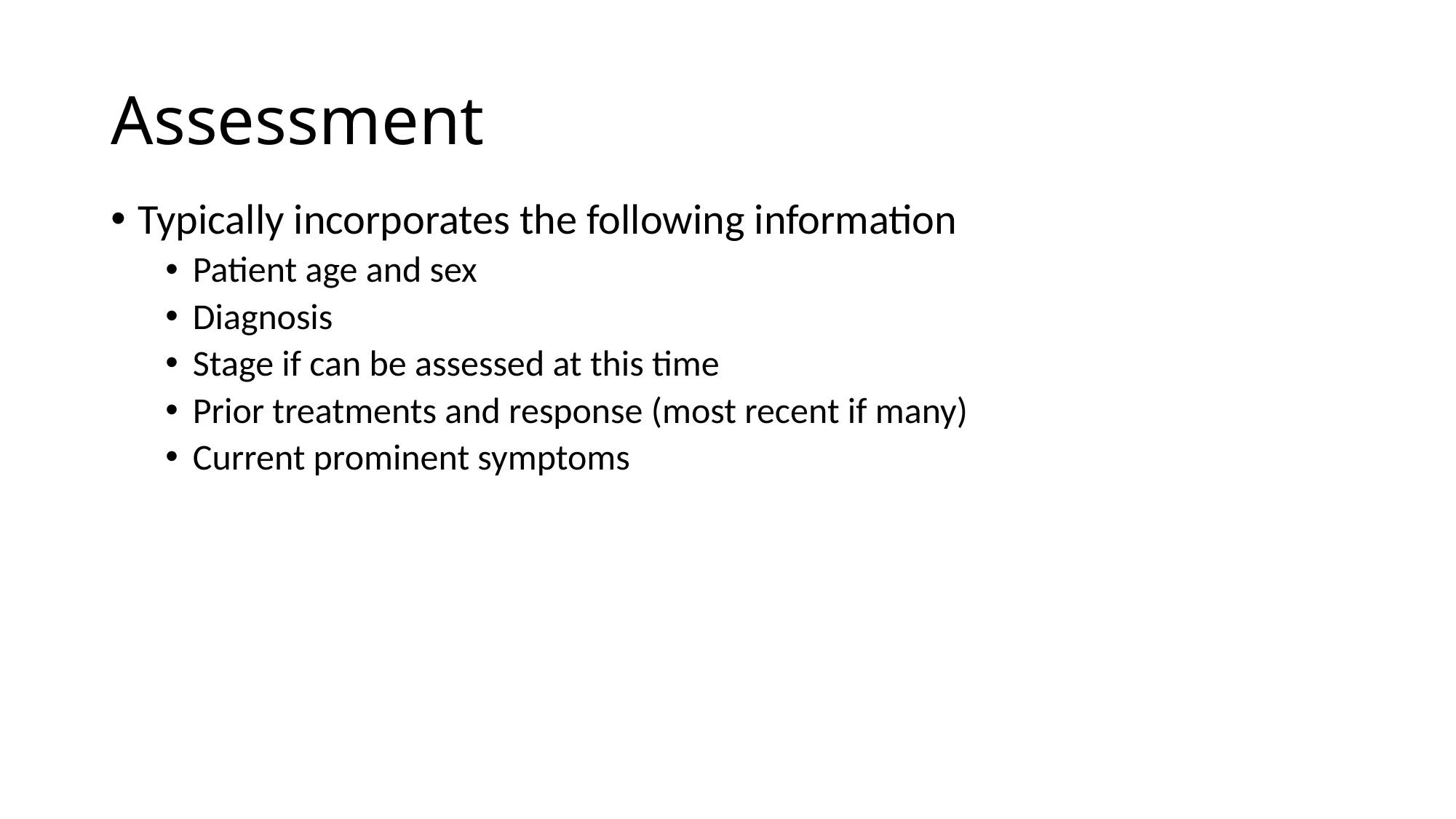

# Assessment
Typically incorporates the following information
Patient age and sex
Diagnosis
Stage if can be assessed at this time
Prior treatments and response (most recent if many)
Current prominent symptoms

## Slide 13
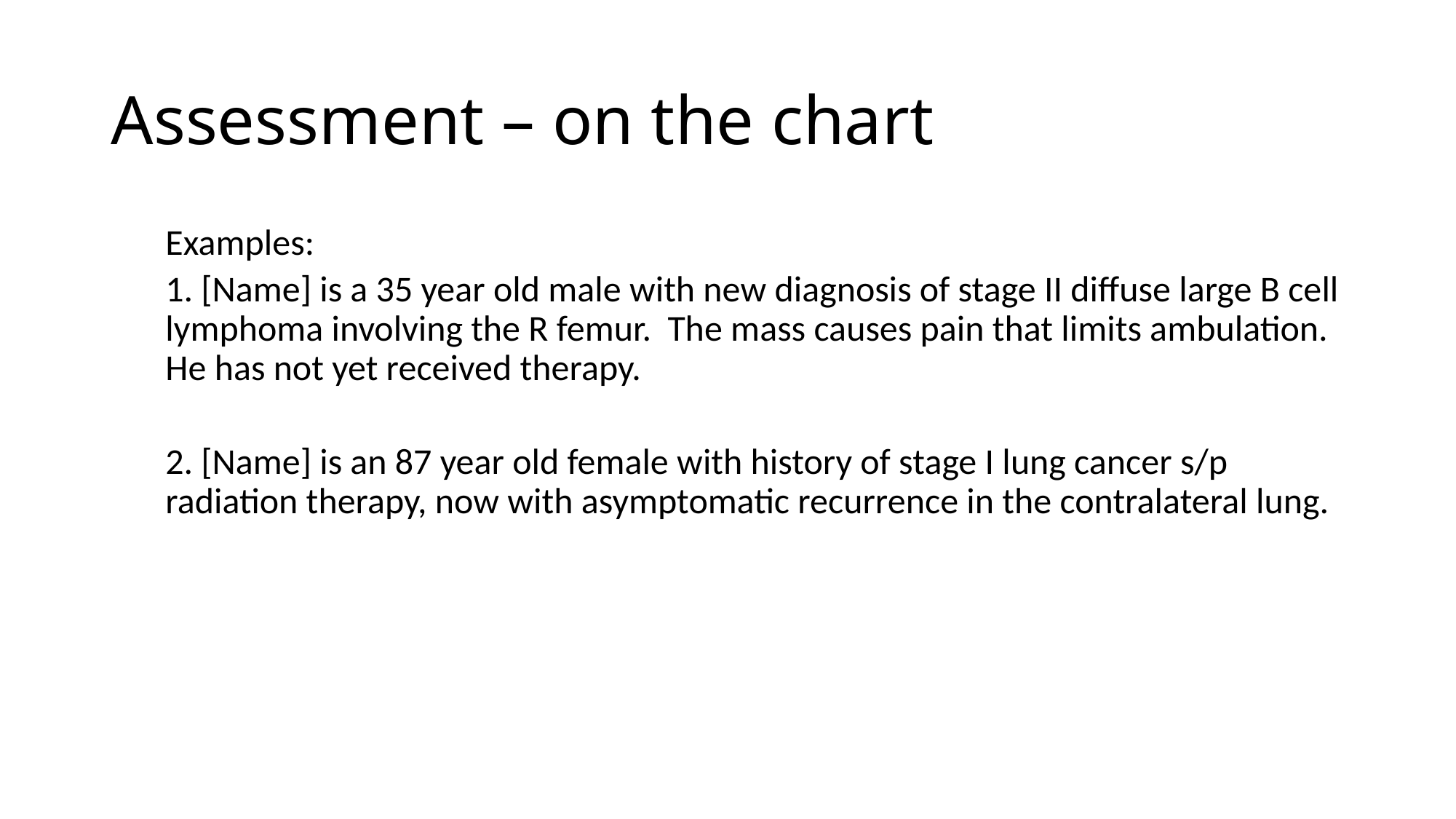

# Assessment – on the chart
Examples:
1. [Name] is a 35 year old male with new diagnosis of stage II diffuse large B cell lymphoma involving the R femur. The mass causes pain that limits ambulation. He has not yet received therapy.
2. [Name] is an 87 year old female with history of stage I lung cancer s/p radiation therapy, now with asymptomatic recurrence in the contralateral lung.

## Slide 14
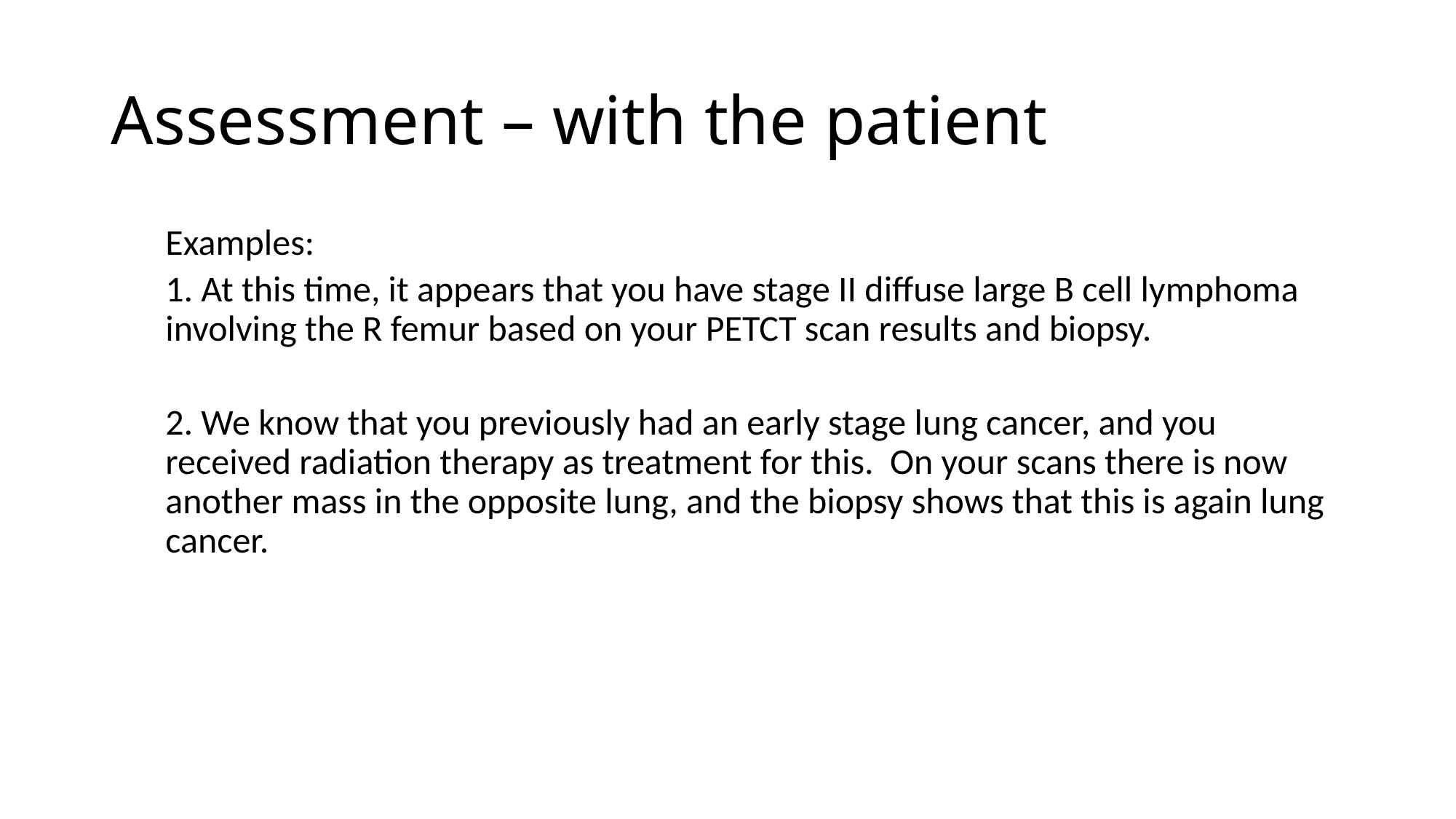

# Assessment – with the patient
Examples:
1. At this time, it appears that you have stage II diffuse large B cell lymphoma involving the R femur based on your PETCT scan results and biopsy.
2. We know that you previously had an early stage lung cancer, and you received radiation therapy as treatment for this. On your scans there is now another mass in the opposite lung, and the biopsy shows that this is again lung cancer.

## Slide 15
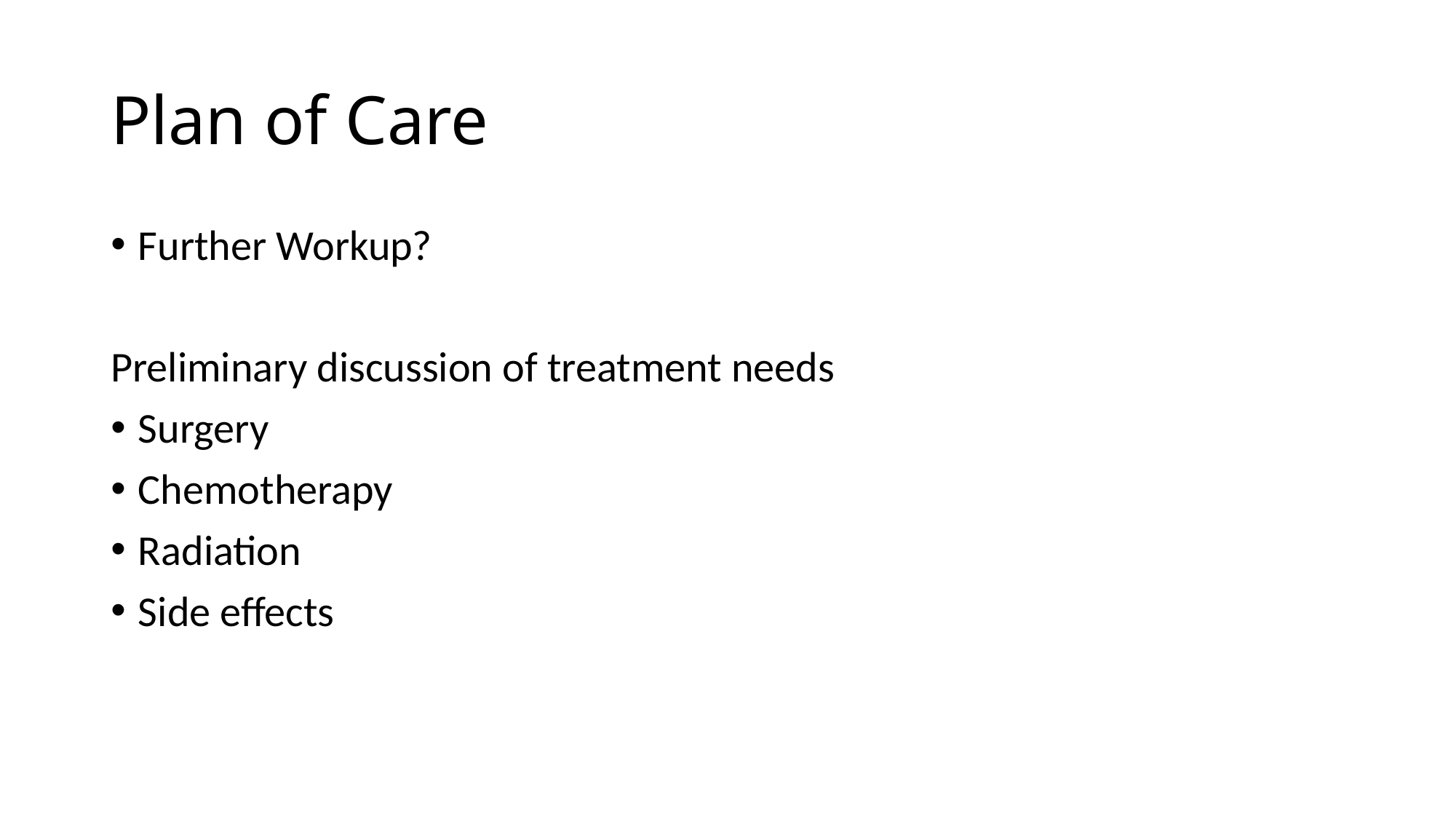

# Plan of Care
Further Workup?
Preliminary discussion of treatment needs
Surgery
Chemotherapy
Radiation
Side effects

## Slide 16
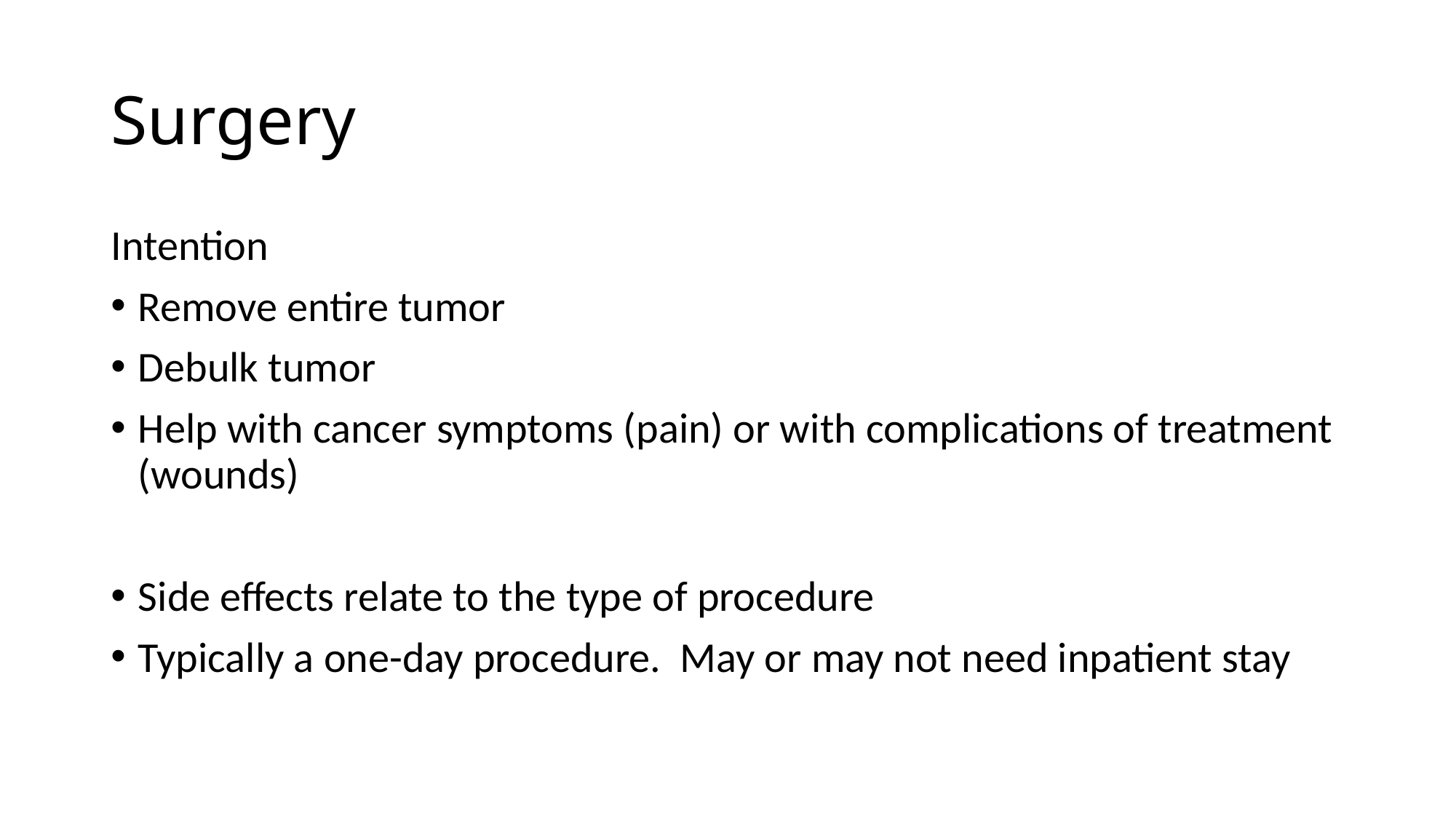

# Surgery
Intention
Remove entire tumor
Debulk tumor
Help with cancer symptoms (pain) or with complications of treatment (wounds)
Side effects relate to the type of procedure
Typically a one-day procedure. May or may not need inpatient stay

## Slide 17
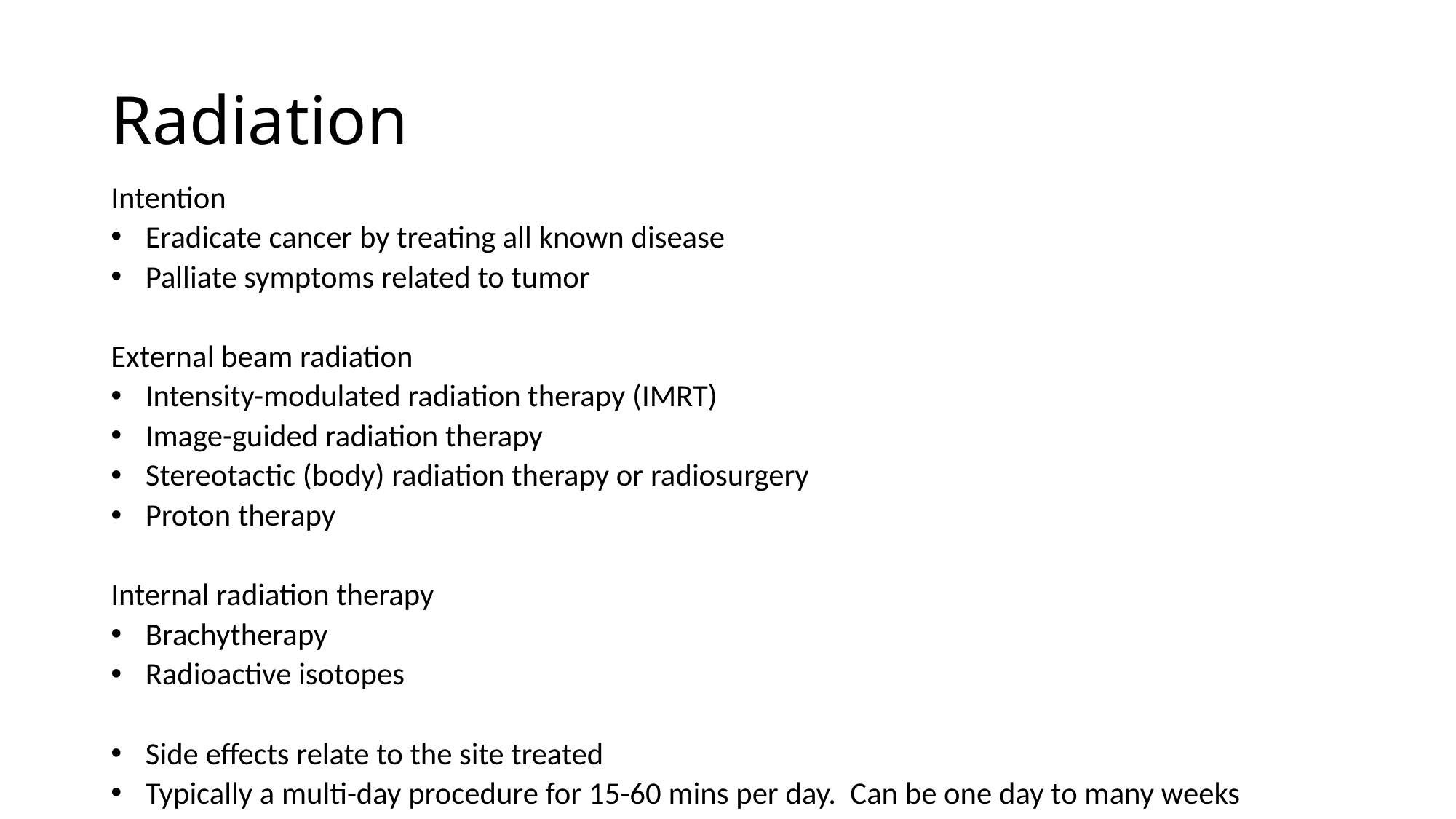

# Radiation
Intention
Eradicate cancer by treating all known disease
Palliate symptoms related to tumor
External beam radiation
Intensity-modulated radiation therapy (IMRT)
Image-guided radiation therapy
Stereotactic (body) radiation therapy or radiosurgery
Proton therapy
Internal radiation therapy
Brachytherapy
Radioactive isotopes
Side effects relate to the site treated
Typically a multi-day procedure for 15-60 mins per day. Can be one day to many weeks

## Slide 18
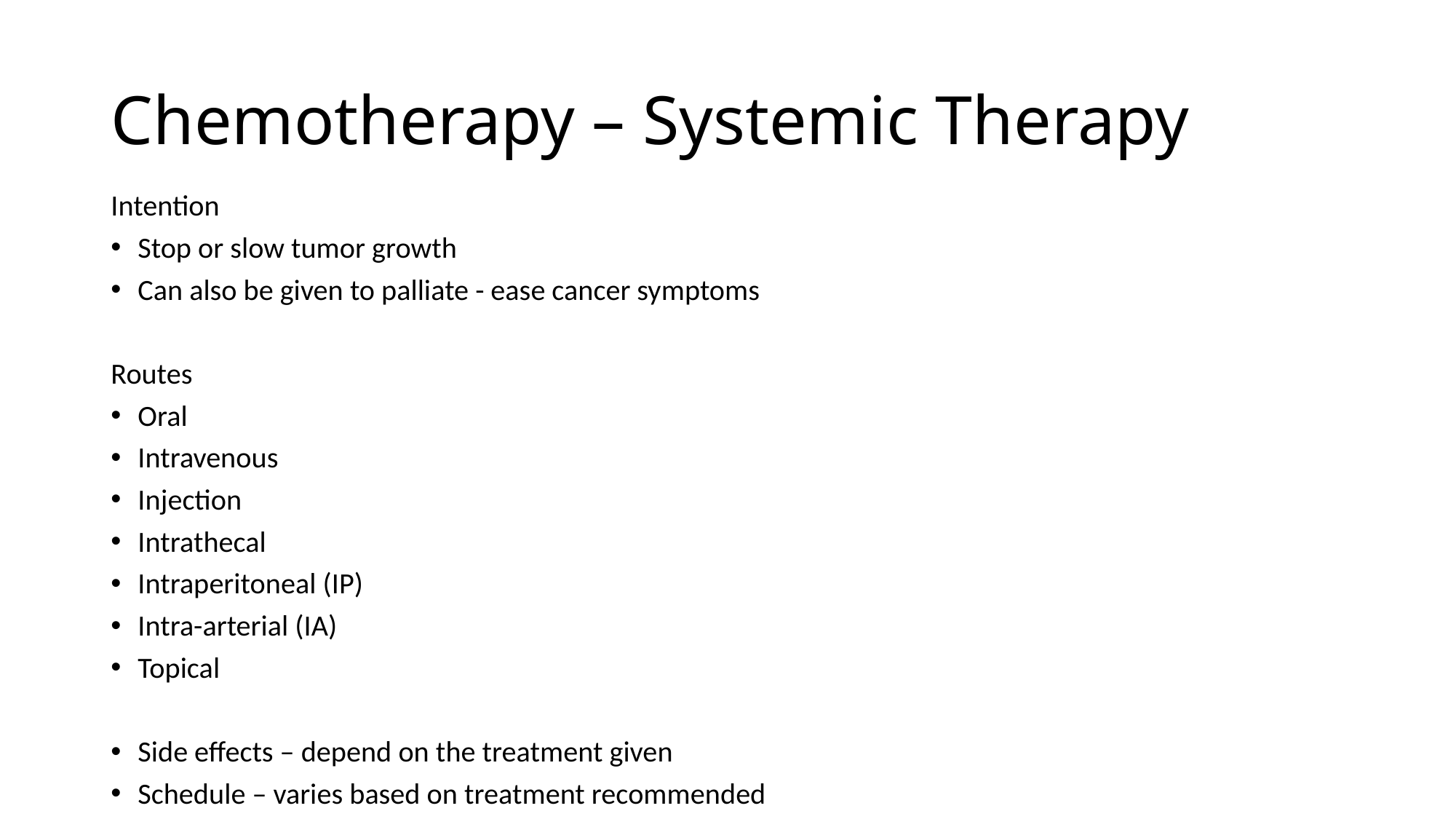

# Chemotherapy – Systemic Therapy
Intention
Stop or slow tumor growth
Can also be given to palliate - ease cancer symptoms
Routes
Oral
Intravenous
Injection
Intrathecal
Intraperitoneal (IP)
Intra-arterial (IA)
Topical
Side effects – depend on the treatment given
Schedule – varies based on treatment recommended

## Slide 19
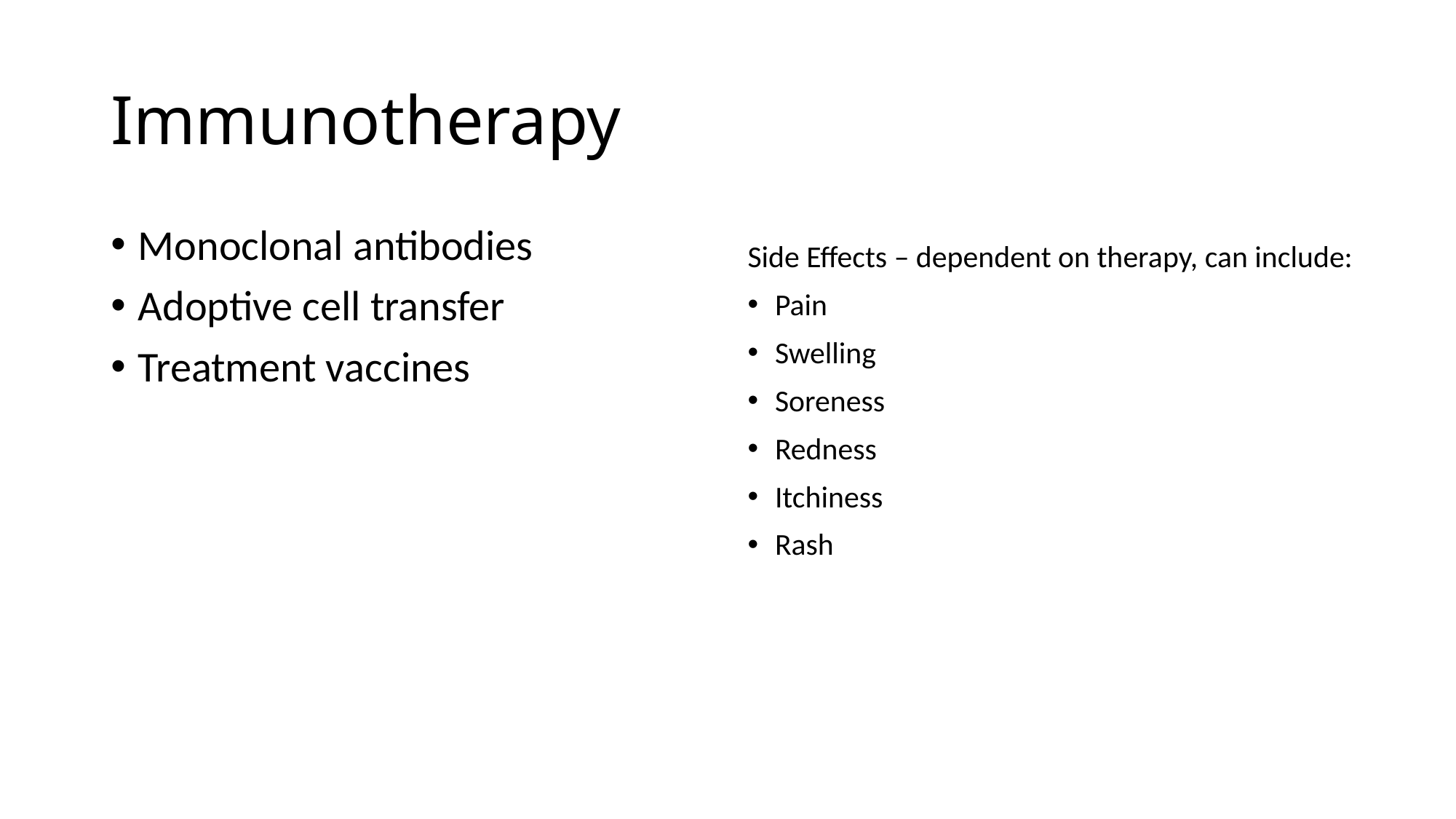

# Immunotherapy
Monoclonal antibodies
Adoptive cell transfer
Treatment vaccines
Side Effects – dependent on therapy, can include:
Pain
Swelling
Soreness
Redness
Itchiness
Rash

## Slide 20
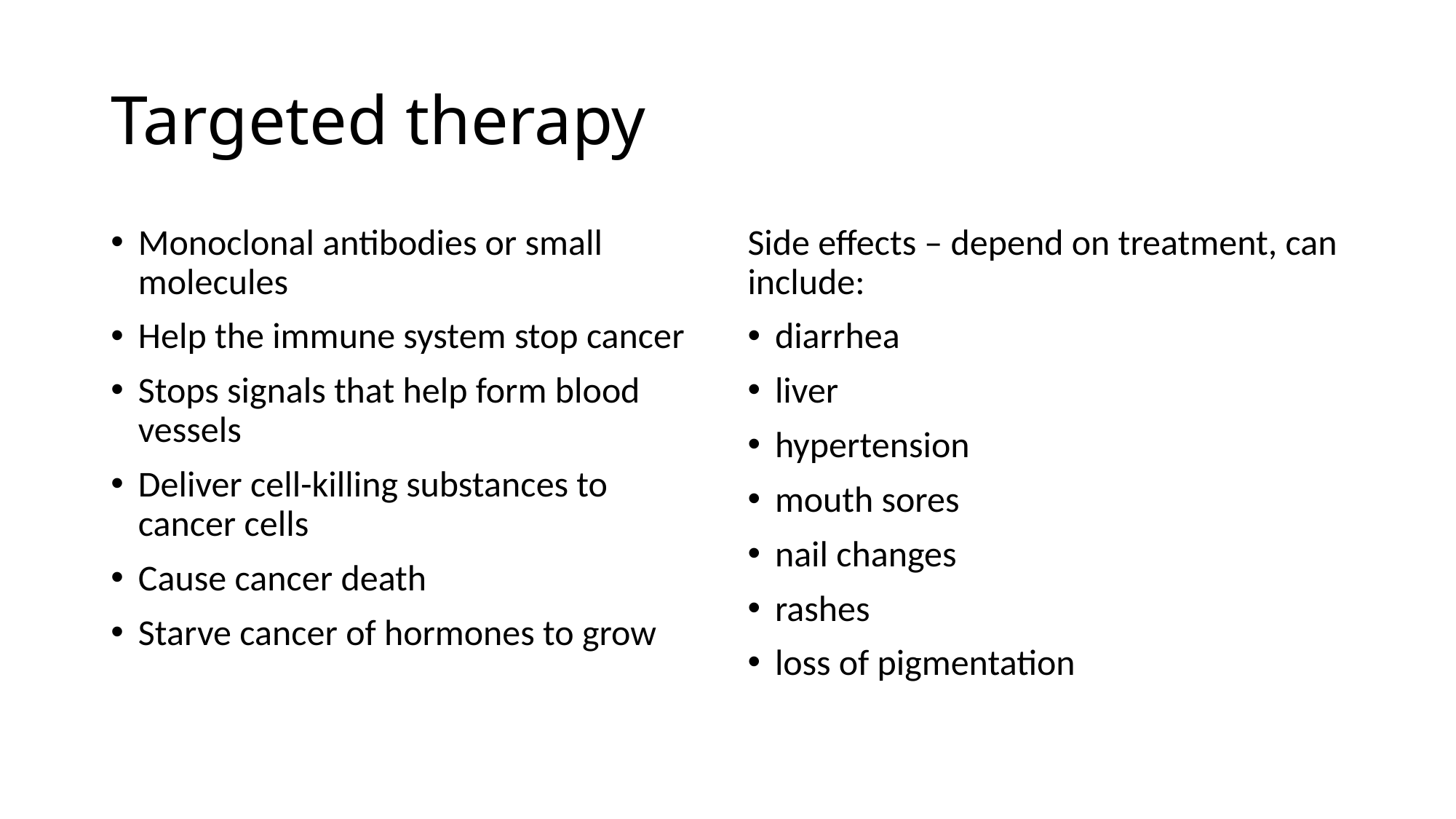

# Targeted therapy
Monoclonal antibodies or small molecules
Help the immune system stop cancer
Stops signals that help form blood vessels
Deliver cell-killing substances to cancer cells
Cause cancer death
Starve cancer of hormones to grow
Side effects – depend on treatment, can include:
diarrhea
liver
hypertension
mouth sores
nail changes
rashes
loss of pigmentation

## Slide 21
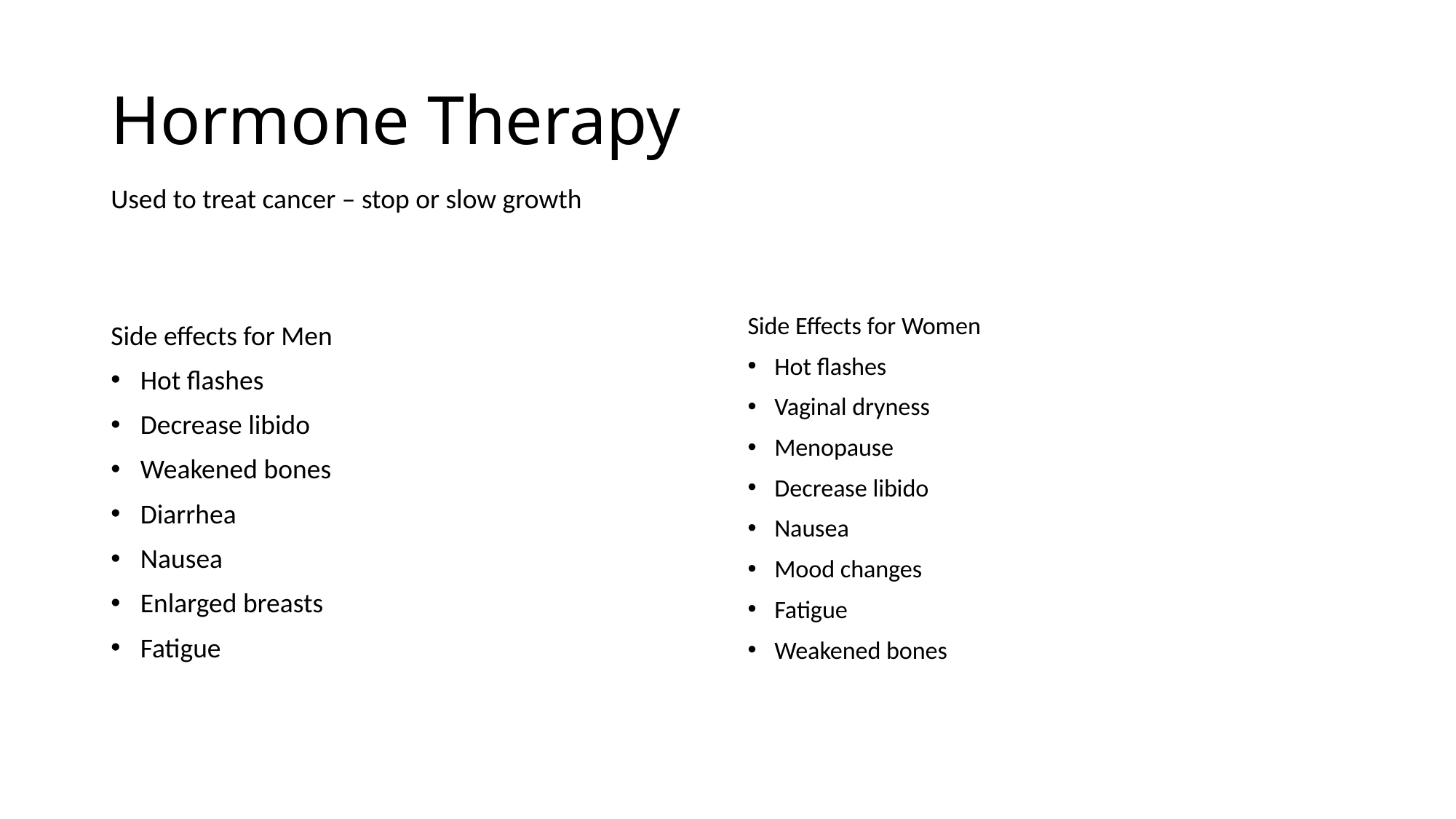

# Hormone Therapy
Used to treat cancer – stop or slow growth
Side Effects for Women
Hot flashes
Vaginal dryness
Menopause
Decrease libido
Nausea
Mood changes
Fatigue
Weakened bones
Side effects for Men
Hot flashes
Decrease libido
Weakened bones
Diarrhea
Nausea
Enlarged breasts
Fatigue

## Slide 22
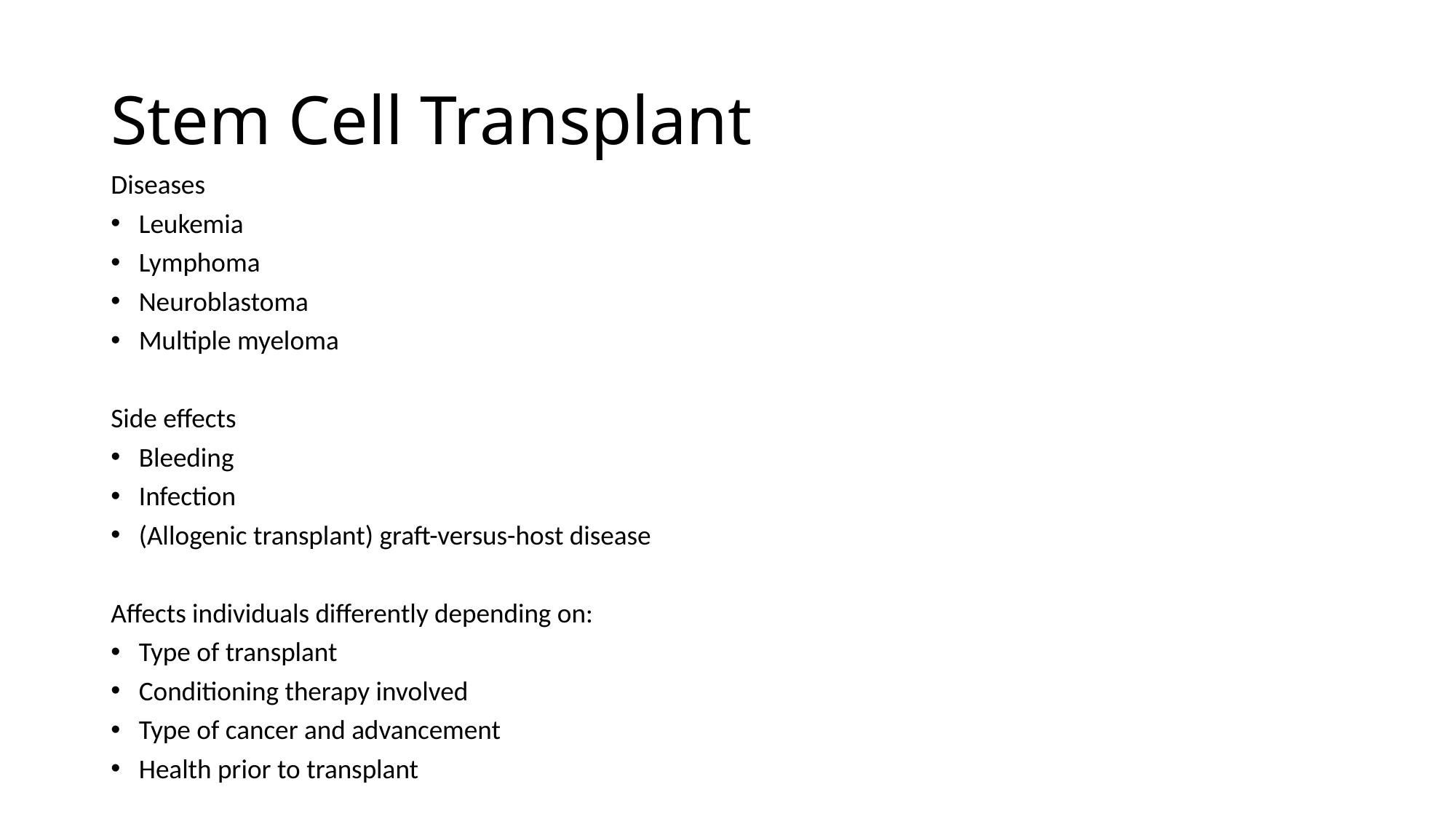

# Stem Cell Transplant
Diseases
Leukemia
Lymphoma
Neuroblastoma
Multiple myeloma
Side effects
Bleeding
Infection
(Allogenic transplant) graft-versus-host disease
Affects individuals differently depending on:
Type of transplant
Conditioning therapy involved
Type of cancer and advancement
Health prior to transplant

## Slide 23
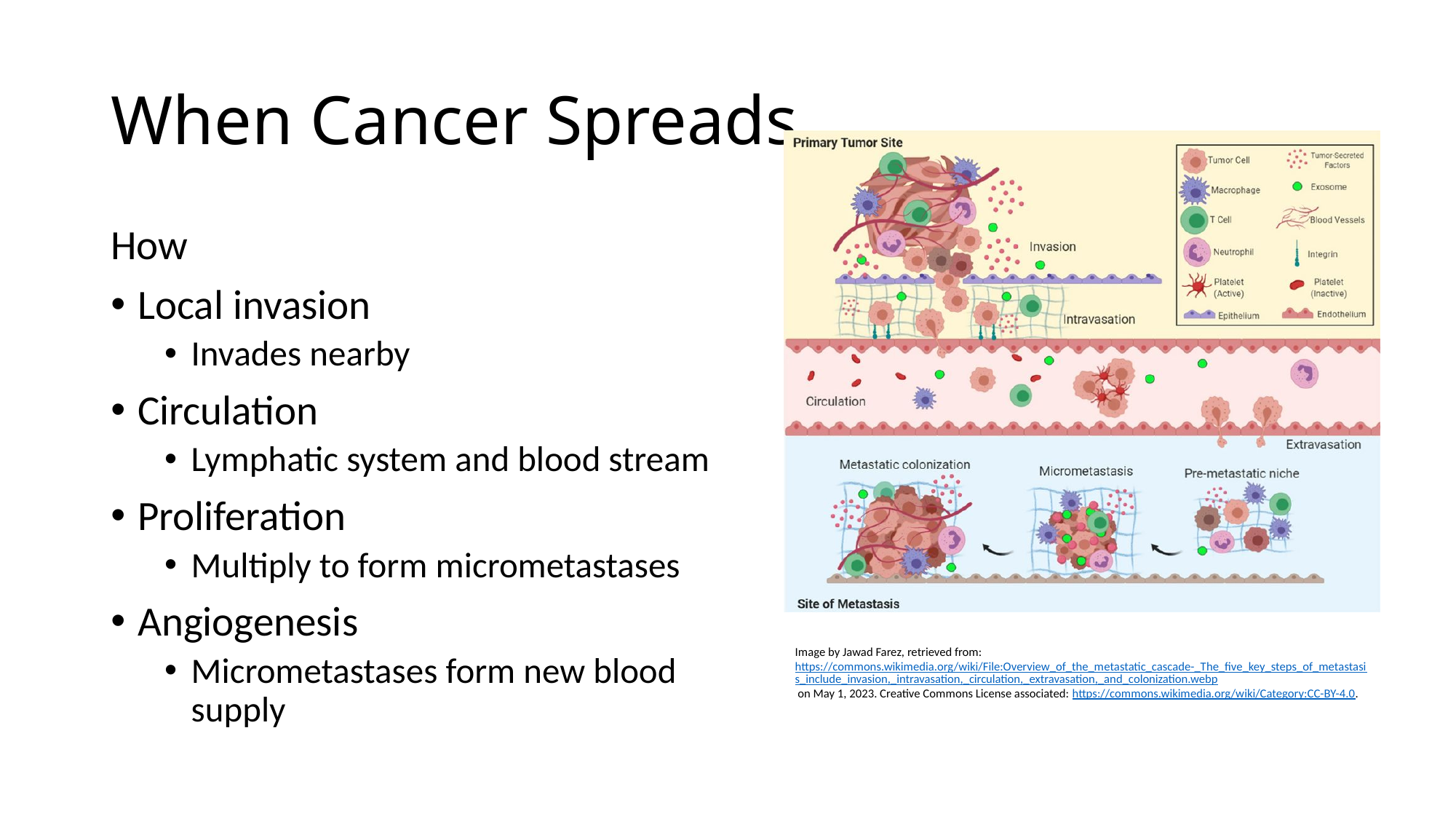

# When Cancer Spreads
How
Local invasion
Invades nearby
Circulation
Lymphatic system and blood stream
Proliferation
Multiply to form micrometastases
Angiogenesis
Micrometastases form new blood supply
Image by Jawad Farez, retrieved from: https://commons.wikimedia.org/wiki/File:Overview_of_the_metastatic_cascade-_The_five_key_steps_of_metastasis_include_invasion,_intravasation,_circulation,_extravasation,_and_colonization.webp on May 1, 2023. Creative Commons License associated: https://commons.wikimedia.org/wiki/Category:CC-BY-4.0.

## Slide 24
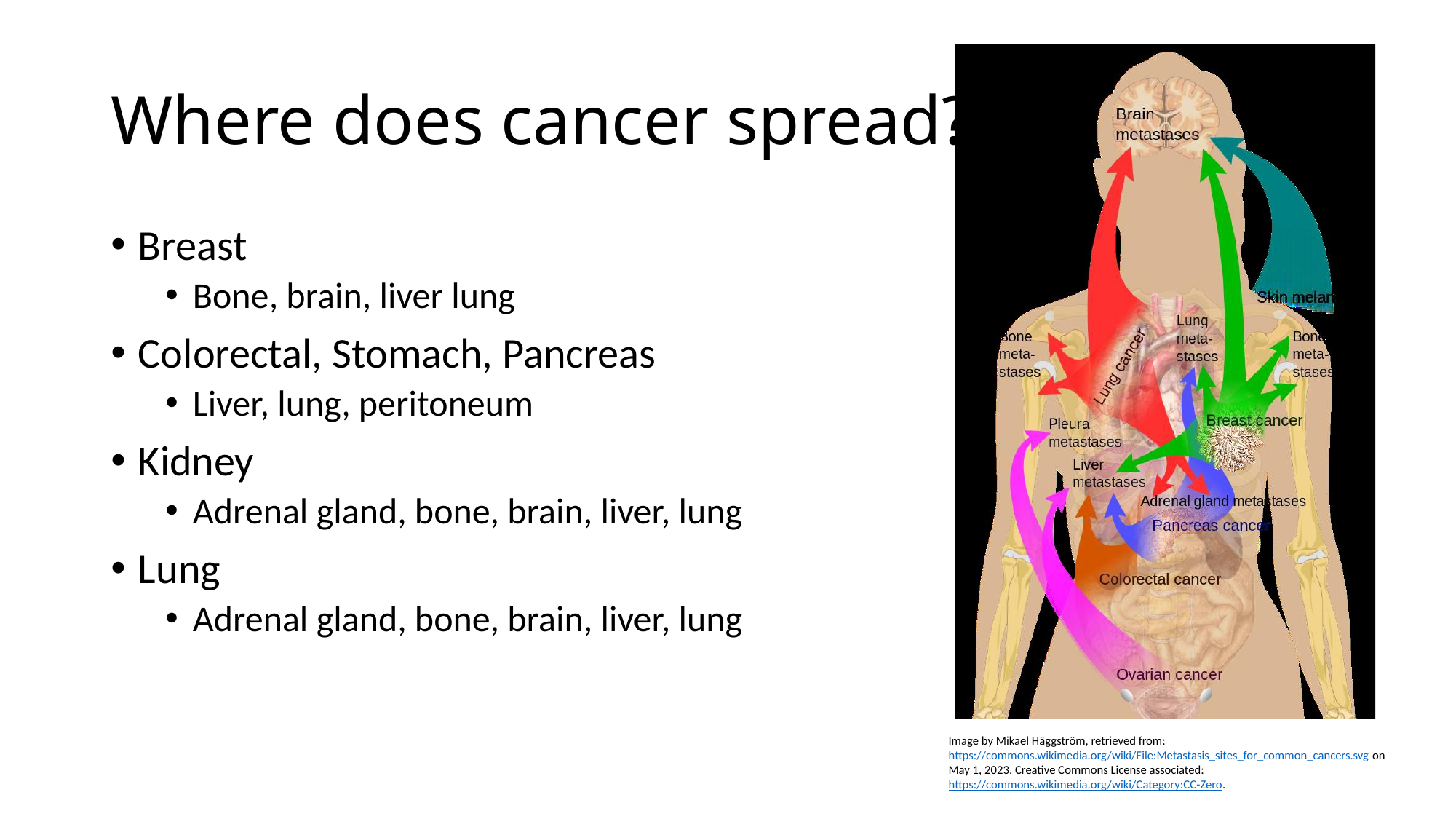

# Where does cancer spread?
Breast
Bone, brain, liver lung
Colorectal, Stomach, Pancreas
Liver, lung, peritoneum
Kidney
Adrenal gland, bone, brain, liver, lung
Lung
Adrenal gland, bone, brain, liver, lung
Image by Mikael Häggström, retrieved from: https://commons.wikimedia.org/wiki/File:Metastasis_sites_for_common_cancers.svg on May 1, 2023. Creative Commons License associated: https://commons.wikimedia.org/wiki/Category:CC-Zero.

## Slide 25
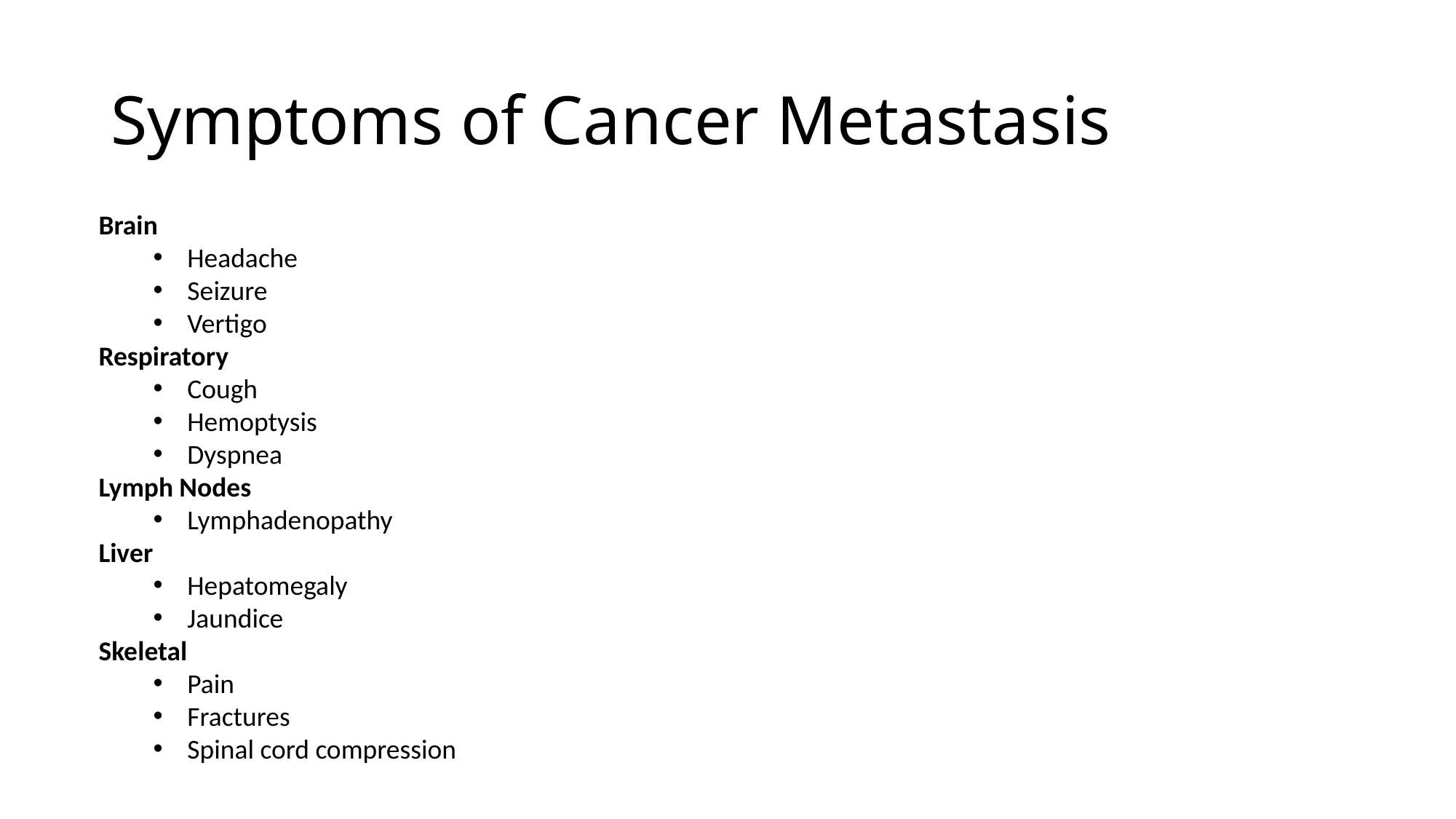

# Symptoms of Cancer Metastasis
Brain
Headache
Seizure
Vertigo
Respiratory
Cough
Hemoptysis
Dyspnea
Lymph Nodes
Lymphadenopathy
Liver
Hepatomegaly
Jaundice
Skeletal
Pain
Fractures
Spinal cord compression

## Slide 26
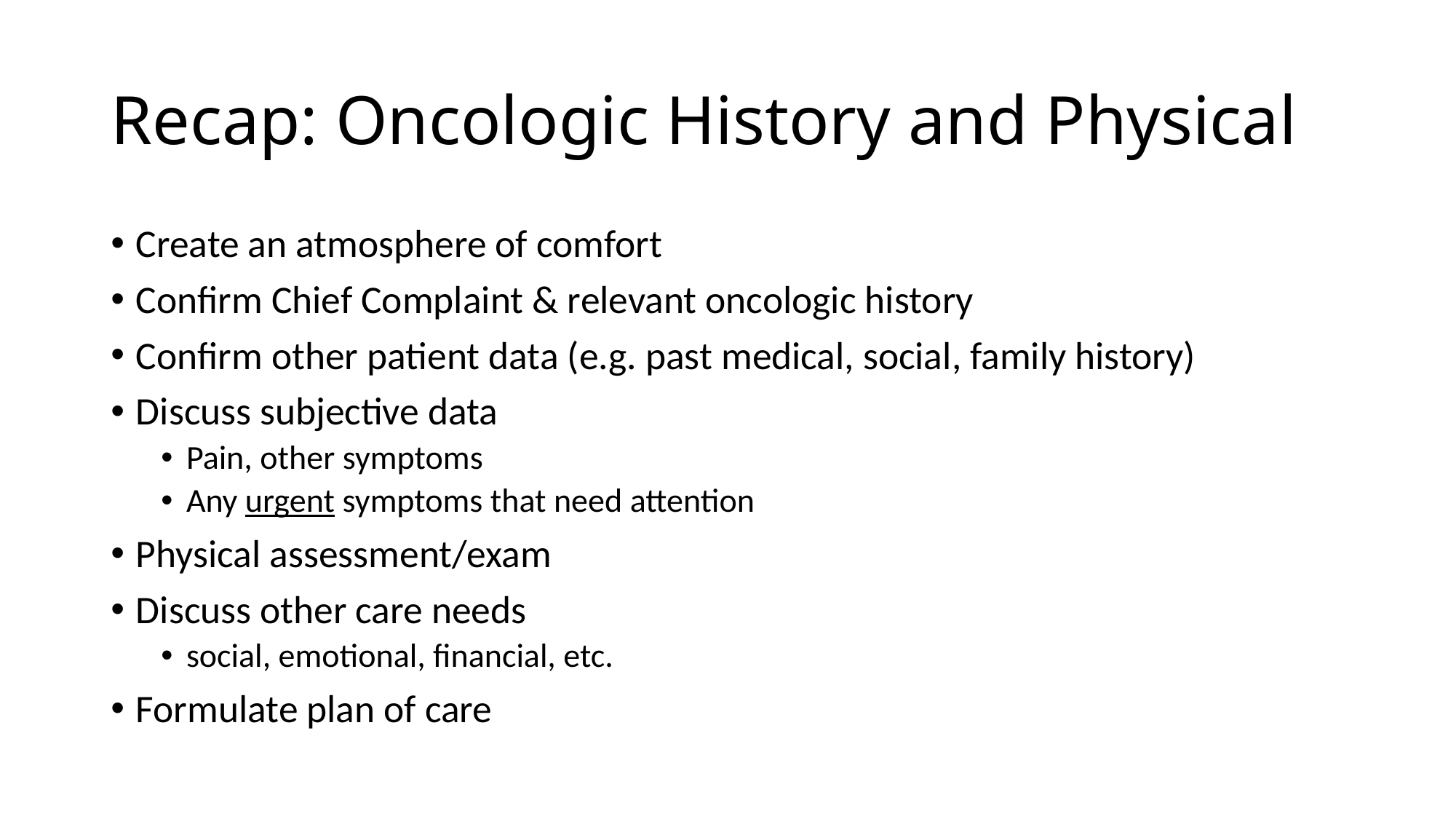

# Recap: Oncologic History and Physical
Create an atmosphere of comfort
Confirm Chief Complaint & relevant oncologic history
Confirm other patient data (e.g. past medical, social, family history)
Discuss subjective data
Pain, other symptoms
Any urgent symptoms that need attention
Physical assessment/exam
Discuss other care needs
social, emotional, financial, etc.
Formulate plan of care

## Slide 27
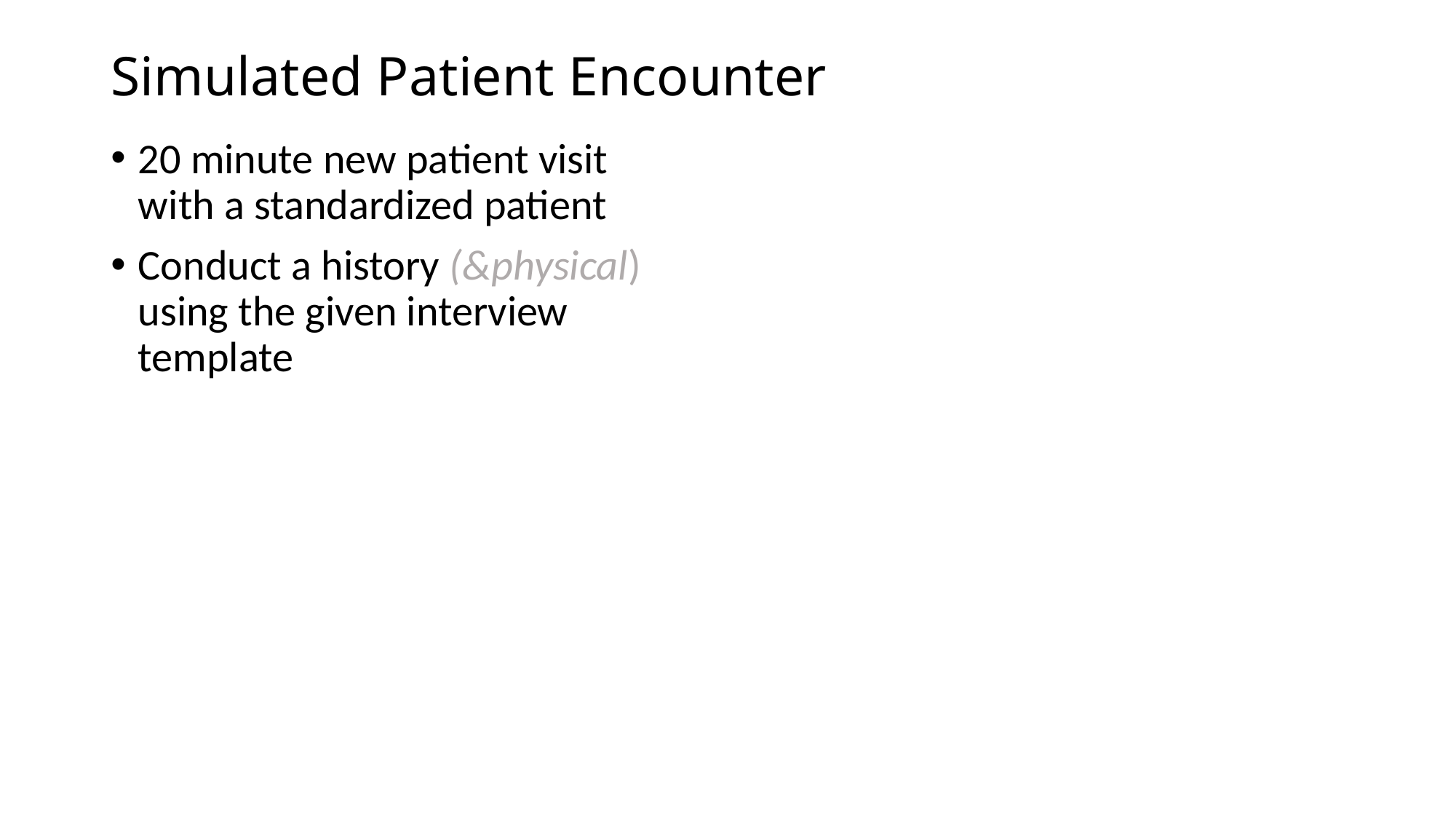

# Simulated Patient Encounter
20 minute new patient visit with a standardized patient
Conduct a history (&physical) using the given interview template

## Slide 28
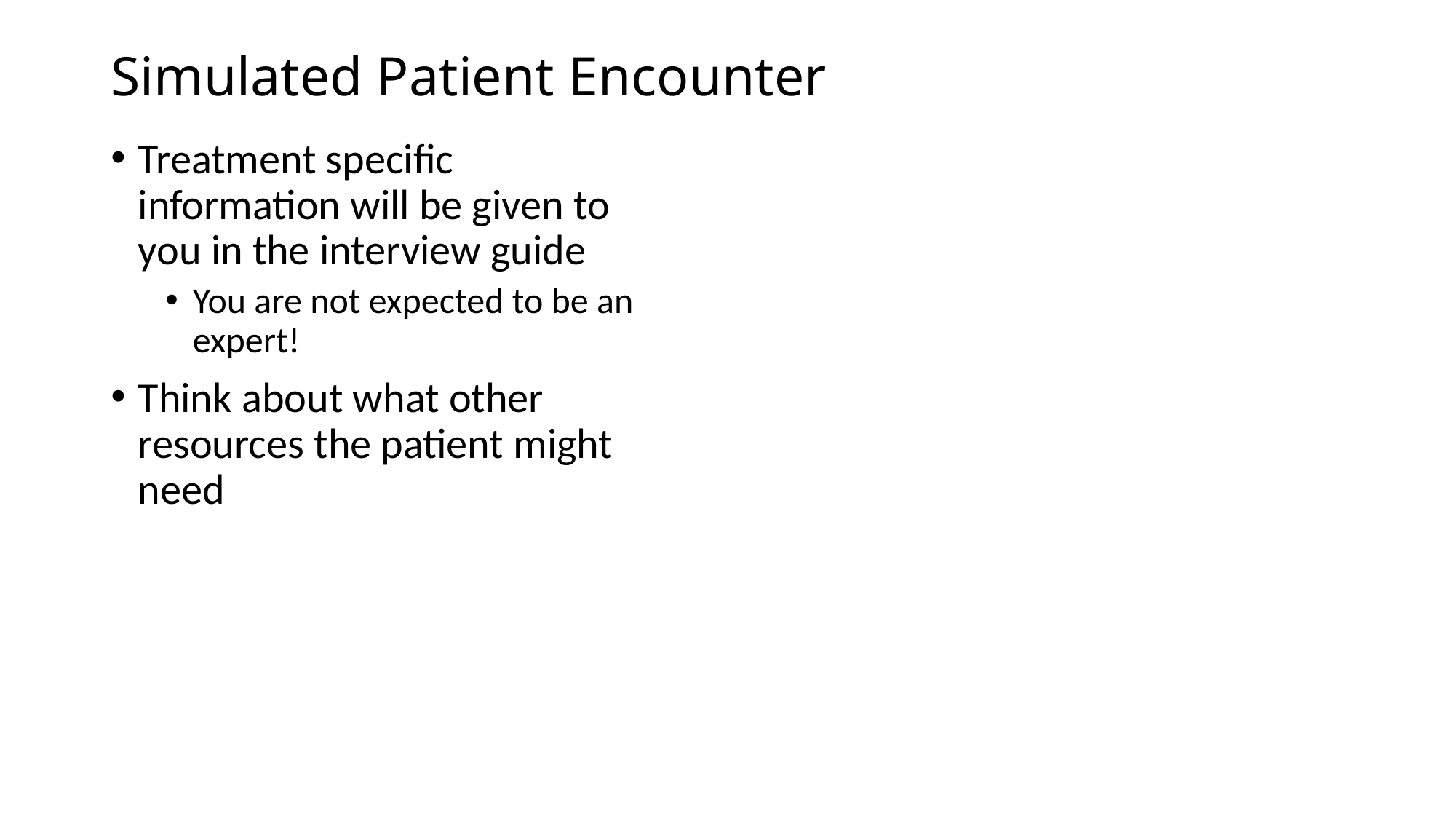

# Simulated Patient Encounter
Treatment specific information will be given to you in the interview guide
You are not expected to be an expert!
Think about what other resources the patient might need

## Slide 29
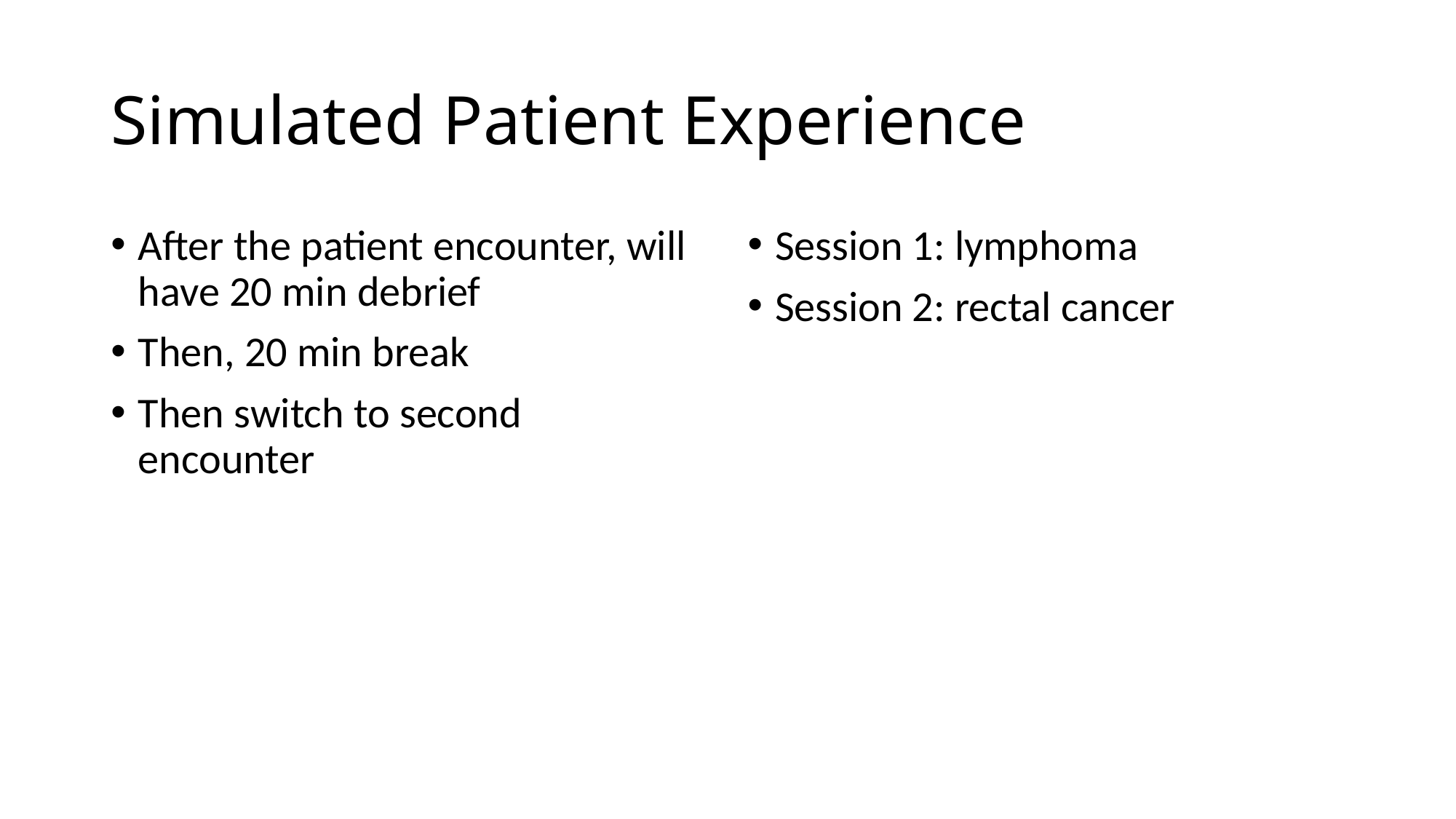

# Simulated Patient Experience
After the patient encounter, will have 20 min debrief
Then, 20 min break
Then switch to second encounter
Session 1: lymphoma
Session 2: rectal cancer
