# Supplementary material for: Approach to an Initial Oncologic Patient Encounter: A Simulation-Based Training for First-Year Medical Students
Source: MedEdPORTAL. 2026 Apr 24;22:11574. doi: 10.15766/mep_2374-8265.11574 (PMC13106612; doi:10.15766/mep_2374-8265.11574)
Supplement: Supplementary file 1 — Approach to an Initial Oncologic Patient Encounter.pptxCase Guide for Students.docxCase Information.docxDebrief Guide for Sim Facilitator.docxPostsimulation Evaluation (Original).docxPostsimulation Evaluation (Revised).docx [file mep_2374-8265.11574-s001.zip › B. Case Guide for Students.docx]

**Case Guide for Students**

**Instructions for Use:** This document is a guide for the learner acting as the provider. It outlines the topic areas that you should consider covering in your patient encounters. Use this guide to structure your interview. It also contains the mock physical exam data and diagnostic results that you must interpret and explain to the patient. Additional information is highlighted below that you might wish to use/observe specifically during each encounter.

There are two distinct cases included here. Ensure you are referencing the correct case for your encounter:

- Lymphoma: Young female patient (Joanne Davis).
- Rectal Cancer: Older male patient (Terry Allen).

**Student Name:**
**Date of Encounter:**
**Clinical Setting (Hospital/Clinic): Lymphoma Clinic**
**Patient's Name (if available): Joanne Davis**
**Age: 25**
**Sex: F**

**1. Presenting Complaint:**

- Chief complaint or reason for seeking medical attention.

**2. History of Present Illness (HPI):**

- Onset and progression of symptoms related to the chief complaint.
  - Ask about onset, setting, duration, timing (frequency, constant), location, radiation, quality, aggravating/relieving factors, associated with?, overall course
- Associated symptoms such as pain, fatigue, weight loss, etc.
- Past workup
- Past treatments and their outcomes.

**3. Past Medical History:**

- Previous medical conditions.
- Surgical history.
- Medications and allergies.

**4. Family History:**

- History of cancer or other relevant diseases among family members.

**5. Social History:**

- Smoking history.
- Alcohol consumption.
- Occupation.
- Living situation.

**6. Review of Systems:**

- General: fever, weight loss, fatigue.
- Constitutional: appetite changes, night sweats.
- Respiratory: cough, dyspnea.
- Gastrointestinal: nausea, vomiting, bowel habits.
- Genitourinary: urinary symptoms, menstrual changes.
- Neurological: headaches, weakness, sensory changes.
- Musculoskeletal: bone pain, joint symptoms.
- Skin: rashes, lesions.

**7. Physical Examination:**

**General:**

- Vital signs: temperature, blood pressure, pulse, respiratory rate, oxygen saturation.
- Vitals:
- Blood Pressure: 135/85
- Pulse: 90
- Temperature: 98.6C
- Respiration: 20
- General appearance: alertness, distress.

**8. Special Investigations (if applicable):**

- Laboratory tests: CBC, comprehensive metabolic panel, tumor markers.
  - Unremarkable except for an ESR of 65
- Imaging studies: X-rays, CT scans, MRI, PET scans.
  - Chest X-ray:
    - There is a large consolidation involving the right lung with a small right pleural effusion. The mediastinum appears widened. Left lung is clear.
    - *[May insert chest X-ray image demonstrating the findings]*
  - Chest CT:
    - 1. Large mass involving the mediastinum, hila, and right lung compatible with malignancy. The mass results in narrowing of the right hilar structures, the brachiocephalic veins, and superior vena cava.
    - 2. Ill-defined groundglass attenuation in the aerated portion of the right lower lobe, which may reflect post-obstructive pneumonitis. Subcentimeter nodules may be infectious, inflammatory, or neoplastic in etiology.
    - 3. Trace right pleural effusion and right lower lobe atelectasis.
    - 4. Enlarged paraesophageal and cardiophrenic lymph nodes.
    - 5. Small pericardial effusion.
    - 6. No CT evidence of pulmonary embolus to the segmental level.
    - *[May insert chest CT image demonstrating the findings]*
- Biopsy results.
  - CT-guided biopsy of the mediastinum:
    - Nodular sclerosis classic Hodgkin lymphoma

**9. Assessment:**

- Summary of findings from history, physical examination, and investigations.
- Explanation of the diagnosis (and prognosis if asked)

So far, we know that you have a diagnosis of Hodgkin Lymphoma that is involving the central portion of your chest and your right lung. We can see this on the chest X-ray and the chest CT scan.

**10. Plan:**

- Further diagnostic workup.
  - We will need additional labs, a PETCT scan, an echocardiogram to evaluate heart function, and pulmonary function tests to evaluate lung function
- Treatment options: surgery, chemotherapy, radiation therapy, immunotherapy, etc.
  - Treatment for lymphoma typically includes systemic therapy and sometimes includes radiation therapy. Surgery is not common. The final plan will be determined once all test results are available.
- Palliative care considerations
- Referrals to other specialists
  - medical oncologist – YES, patient will need systemic therapy
  - surgeon
  - palliative care team
  - physical/occupational/speech therapy
  - nutrition
  - fertility – YES, patient is interested in having children.
  - pulmonology – Maybe. will need to watch lung function and shortness of breath carefully. Treatment initiation will need to be expedited and she should come to the emergency room for any worsening symptoms.
  - Social work – YES, patient has concerns related to time off work
  - Mental health – YES, good to ask if patient needs any related resources
- Follow-up arrangements.
  - We will get the additional testing done and the referrals to other providers. I’ll plan to see you back to discuss all the results in ~2 weeks.

**11. Patient Education:**

- Addressing patient concerns and questions

**Student Name:**
**Date of Encounter:**
**Clinical Setting (Hospital/Clinic): GI Cancer Clinic**
**Patient's Name (if available): Terry Allen**
**Age: 55**
**Sex: M**

**1. Presenting Complaint:**

- Chief complaint or reason for seeking medical attention.

**2. History of Present Illness (HPI):**

- Onset and progression of symptoms related to the chief complaint.
  - Ask about onset, setting, duration, timing (frequency, constant), location, radiation, quality, aggravating/relieving factors, associated with?, overall course
- Associated symptoms such as pain, fatigue, weight loss, etc.
- Past workup
- Past treatments and their outcomes.

**3. Past Medical History:**

- Previous medical conditions.
- Surgical history.
- Medications and allergies.

**4. Family History:**

- History of cancer or other relevant diseases among family members.

**5. Social History:**

- Smoking history.
- Alcohol consumption.
- Occupation.
- Living situation.

**6. Review of Systems:**

- General: fever, weight loss, fatigue.
- Constitutional: appetite changes, night sweats.
- Respiratory: cough, dyspnea.
- Gastrointestinal: nausea, vomiting, bowel habits.
- Genitourinary: urinary symptoms, menstrual changes.
- Neurological: headaches, weakness, sensory changes.
- Musculoskeletal: bone pain, joint symptoms.
- Skin: rashes, lesions.

**7. Physical Examination:**

**General:**

- Vital signs: temperature, blood pressure, pulse, respiratory rate, oxygen saturation.
- Vitals:
- Blood Pressure: 120/80
- Pulse: 80
- Temperature: 98.6C
- Respiration: 14
- General appearance: alertness, distress.

**8. Special Investigations (if applicable):**

- Laboratory tests: CBC, comprehensive metabolic panel, tumor markers.
  - Unremarkable except for Hemoglobin of 9.5 g/dL (LOW)
- Imaging studies: none
- Colonoscopy report:
  - There is a multilobular polypoid mass located in the proximal rectum extending distally 5cm
- Biopsy results.
- Pathology: Biopsies, rectal mass: Invasive moderately differentiated adenocarcinoma, HPV negative

**9. Assessment:**

- Summary of findings from history, physical examination, and investigations.
- Explanation of the diagnosis (and prognosis if asked)

From the results of the colonoscopy, we know that you have a diagnosis of rectal cancer. Your blood counts (hemoglobin) are low, probably related to bleeding from the tumor.

**10. Plan:**

- Further diagnostic workup.
  - We will need additional labs and imaging to determine the best treatment plan. Oftentimes, we need to repeat a special kind of study called proctoscopy to better determine the extent of the tumor and what treatment is best.
- Treatment options: surgery, chemotherapy, radiation therapy, immunotherapy, etc.
  - Treatment for rectal cancer can include surgery, radiation, and systemic therapy, depending on the stage of the cancer and the results of the remaining tests.
- Your hemoglobin is low. If you notice increased bleeding or dizziness/lightheadedness, chest pain, shortness of breath, you should come to the emergency room. Also, if you cannot pass regular bowel movements, you should come to the emergency room.
- Palliative care considerations
- Referrals to other specialists
  - medical oncologist – YES, patient may need systemic therapy
  - surgeon – YES, patient may need surgery
  - palliative care team
  - physical/occupational/speech therapy
  - nutrition
  - fertility/sexual activity – YES, patient is sexually active and treatment could affect sexual function
  - Social work and billing department – YES, patient has concerns related to time off finances
  - Mental health – YES, good to ask if patient needs any related resources
- Follow-up arrangements.
  - We will get the additional testing done and the referrals to other providers. I’ll plan to see you back to discuss all the results in ~2 weeks.

**11. Patient Education:**

- Addressing patient concerns and questions
