# Supplementary material for: Approach to an Initial Oncologic Patient Encounter: A Simulation-Based Training for First-Year Medical Students
Source: MedEdPORTAL. 2026 Apr 24;22:11574. doi: 10.15766/mep_2374-8265.11574 (PMC13106612; doi:10.15766/mep_2374-8265.11574)
Supplement: Supplementary file 1 — Approach to an Initial Oncologic Patient Encounter.pptxCase Guide for Students.docxCase Information.docxDebrief Guide for Sim Facilitator.docxPostsimulation Evaluation (Original).docxPostsimulation Evaluation (Revised).docx [file mep_2374-8265.11574-s001.zip › C. Case Information.docx]

Case Information (SP Scripts)

Instructions for Use: This document contains the detailed information for the Standardized Patient (SP) actors.

For the SP actors, this script dictates your "Symptom Story" and your specific emotional reactions. You must memorize the specific history provided in the "Patient Profile" to answer student questions accurately.

For the Facilitator, use this to verify that the SP is delivering accurate information and to understand the "hidden" concerns (e.g., fertility, financial strain) the student is expected to uncover.

SP Case: Lymphoma Patient

Case Adapted from: Adkins LD, Harris BS, Gesher C, Reynolds T, Branford K, Baldwin M, Dotters-Katz S. Understanding Patient Evaluation of Abnormal Uterine Bleeding (AUB): A Standardized Patient Case on AUB for OB/GYN Clerkship Students. MedEdPORTAL. 2022 Jan 28;18:11216. doi: 10.15766/mep_2374-8265.11216. PMID: 35136836; PMCID: PMC8795174.

Patient Name: Joanne Davis (she/her/hers)

Chief Complaint: “I have a cough”

Type and level of learner: first/second year medical student

Case Objectives:

1. Gather an oncology-focused history for a patient with new cancer diagnosis
2. Assess patient needs including urgent/emergent medical care, multidisciplinary referrals, emotional/spiritual needs, interprofessional evaluations

**Suggested Time for Components (Total Encounter Length 20 minutes):**

Introduction: 2 minutes

History of Present Illness: 6 minutes

Review of Systems: 3 minutes

Physical Exam: N/A

Review of imaging and completed workup: 3 minutes

Assessment and Plan: 6 minutes

| SETTING: outpatient, in patient, ED, home, nursing home, rehab, group etc. | outpatient |
| --- | --- |
| PATIENT PROFILE: Information about the “patient” that helps select an SP and helps the learner get an understanding of them as a person. SP will know more information about the patient than learner will ever ask but allows SP to portray a fully developed patient personality. If none of the items below are particulars for the case please write “all may be used.” | |
| Age range | 25-40 (stated age 25) |
| Race and/or ethnic group | All may be used |
| Religious/spiritual background | All may be used |
| Sex (e.g., male, female, intersex, transwoman, transman) | female |
| Sexual Orientation (e.g., heterosexual, lesbian, gay, bisexual, pansexual, queer, asexual) | heterosexual |
| Gender expression (e.g., man, woman, gender queer) | woman |
| Race/ethnicity: | All may be used |
| Physical description (e.g., BMI, height range) | All may be used |
| Physical limitations (e.g.,) |  |
| Patient appearance (e.g., disheveled, hospital gown, business casual, casual) | Hospital gown, well-groomed. |
| Moulage + location (e.g., none, bruises, scars, body piercing, tattoos) | none |
| Affect (e.g., pleasant, cooperative) | You are not very concerned about the diagnosis. You are cooperative with student. |
| Family group (e.g., who is family, who they live with) | Lives with husband |
| Education | High school diploma |
| Level of health literacy | Middle—is familiar with anatomical words such as lymph node |
| Employment, if any - present and past, noting any current stresses | Works at WalMart |
| Home/homeless - type of dwelling, number of stories, owned or rented | Rents a 2-bedroom, 2-bathroom home |
| Financial situation- any current stresses | Low income, but no current acute stresses |
| Insurance Status (e.g., un/under/insured, public/private, HMO/PPO) | Insured through her work |
| Habits (i.e., diet, exercise, caffeine, smoking, alcohol, drugs) | Tobacco: Denies  Alcohol, drugs, substances: Does not use drugs. Uses alcohol only on special occasions, 1-2x/month  **If asked – all alcohol abuse related questions negative  Diet: Eats fast food a lot, tries to add in vegetables, but doesn’t really like them.  Exercise: None |
| Activities (i.e., hobbies, sports, clubs, friends) | Any |
| Typical day - what is the usual daily routine | Wakes up between 6-7am, breakfast generally on the go, arrives to work at 8 am. Returns home after 5:30pm. She eats dinner with her husband (most nights fast food) and they usually watch a TV show before going to bed. |

| CASE INFORMATION | |
| --- | --- |
| Chief Concern: What the patient will say when greeted by the student. The patient’s primary reason for seeking medical care often stated in his/own words. | “I have been coughing a lot and feeling short of breath. Urgent care said I needed a chest X ray and then they made me go to the ER and stay for a biopsy and observation before they would let me go home.” |
| Additional Concerns: Other, if any, concerns the patient has today (i.e., symptoms, requests, expectations, etc.) that will become part of set agenda. | None |
|  | |
| THE PATIENT STORY: The SP will be asked to tell their symptom story and the personal and emotion impact for each of their concerns. You will want to write this is the patient voice. The symptom story should be able to answer this question:“Tell me more about [chief concern/additional concern], starting at the beginning and bringing me up to now.”  The personal context should be able to answer questions concerning the broader personal/psychosocial context of symptoms, especially the patient beliefs/attributions.  The emotional context should be able to ask how are you doing with this, how does this make you feel, how has this affected you emotionally? IMPACT: How has this affected your life? How has this been for your family? | “Well, I really just thought I had a cold. I’m around so many people at work. I started with this cough and maybe a runny nose. But then a few weeks went by and the cough was just getting worse. So I stopped at urgent care and they gave me some antibiotics maybe a few weeks ago, but I didn’t get any better. I went back to urgent care again and they said that I needed a chest X ray so I went to the emergency room and they told me that it looks l have a large tumor in my chest. They were worried about my ability to breathe so they kept me and did a biopsy there in the hospital. They told me that it’s lymphoma. They said that I have to see someone really soon.  I know that I need to be here, but really I’m supposed to be at work and my boss is not very understanding. Do you know how long this appointment is going to take?” |
| HISTORY OF PRESENT ILLNESS: Although some of the HPI will be given in the patient’s symptom story, the learners will expand the story during the direct question section. Below describe the detailed history, usually about the chief concern, which the student must develop in order to make a useful assessment of the problem: | |
|  | |
| Onset (when; gradual or sudden) | This cough started about two months now. I have been a little short of breath when doing heavy activity at work for maybe the past month. |
| Setting (what was going on or where was patient when symptoms first noticed?) | I first thought I had a cold one night when I couldn’t stop coughing while watching tv at home. |
| Duration (how long) | The cough is constant and really irritating. I don’t cough up anything. It feels like something is irritating my chest and I just have to cough. It’s worse after any activity and when I’m active I also feel pressure in my chest and like it’s hard to breath. But I pretty much have a cough all the time. |
| Time relationships (frequency, constant or intermittent) |  |
| Location | My chest |
| Radiation | None |
| Quality | It’s horrible for my quality of life. I feel like everyone at work is looking at me like I’m sick and contagious. I can’t do all the activities that I’m supposed to do at work. |
| Amount |  |
| Aggravated by what | Activity |
| Relieved by what | If I sit quietly it seems to be better. Cough medicines haven’t helped and the antibiotics only improved things for about a week. |
| Associated with what | Activity |
| Attitude (what does the patient think is the problem, and how does he/she feel about it) | The ongoing coughing is really bothersome. It seems like so much sometimes; I did wonder if it might be something serious. |
| Overall course | I’ve been coughing for about two months. I saw urgent care about a month ago. The cough, chest pressure, and shortness of breath has continued to gradually worsen over this time. The past week, I’ve woken up in the middle of the night really sweaty. I guess the weather has been warmer and I’m not used to it. |
| REVIEW OF SYSTEMS: Significant positives and negatives | |
| *Negative* | *Positive* |
| No fevers or chills | Dry cough |
| No itching or rash | Shortness of breath, especially with activity |
| No neck or face swelling | Chest pressure |
| No bleeding or coughing up blood | Positive for weight loss – unsure of amount |
| No new lumps or bumps | Night sweats |
|  |  |
|  | |
| Past medical history |  |
| Medication allergies (Name and reaction) | None |
| Environmental allergies (Name and reaction) | None |
| Illnesses | None |
| Vaccinations | Up to date, had flu shot this year |
| Surgeries | None |
| Accidents/ injuries/ trauma | None |
| Hospitalization |  |
|  | |
| Inclusive sexual and reproductive history | |
| Sexual practices  Sexual partners  Protection: Use of safer sex practices  Use of birth control if appropriate  Risk of intimate partner violence | Vaginal and oral sex;  Currently 1 male partner, lifetime 3 male partners  None, “It’s just me and my husband”  Never used hormonal birth control, only condoms.  None, no domestic violence no sexual abuse |
| Ob/GYN HISTORY | Age of onset of menses: 10  Periods: Used to be regular, every 28 days, lasting 5-6 days, heavy first 3 days.  Age of menopause: N/A, mother went through menopause at ~age 53  Pap smears: 1 abnormal Pap in 2005, but they have been ok since. Pap smear last year was normal and the additional testing (HPV) was negative.  Hx of STDs: Chlamydia as a teenager.  Number of pregnancies: 0  Number of live births: 0  Number of miscarriages: 0  Number of abortions: 0 |
| Medications | Prescription/dose/reason: None  Over the counter/dose/reason: Motrin PRN/600mg/cramps  Herbs/supplements/dose/reason: None  Other: None |
| Immunizations | X Tetanus  X Flu  X Hepatitis   - Pneumovax - HPV - Other |
| Tobacco products:   - Cigarettes - Cigar - Pipe - Chew - E-cigarettes | X Never   - Past- year started/year quit |
| Alcohol   - Beer - Wine - Liquor - Other | - Never - Past- year started/year quit   X Current  Quantity: 1-2 glasses of wine month/special occasions  # of years: Past 4 years |
| Drugs   - Weed - Cocaine - Heroin - Meth - Other - IV - Inhalants - Other | X Never   - Past- year started/year quit - Current   - Quantity - # of years |
| Diet (describe) | Primarily fast food, tries to add in vegetables, but doesn’t really like them. |
| Exercise (describe) | See Habits |
| List any other important social history or information important to this case |  |
| Family history |  |
| Mother, Father, Siblings, Grandparents, and other significant findings. | Family member, status (living age/deceased), medical conditions  • Father, living 60 years old, type 2 diabetes, hypertension  • Mother, living 62 years old, type 2 diabetes  • Sibling, living 40 years old, Hypertension  • Children, none |
|  |  |
| Physical Exam- Deferred  Chest X-ray:  There is a large consolidation involving the right lung with small right pleural effusion. The mediastinum appears widened. Left lung is clear.  Chest CT:  1. Large mass involving the mediastinum, hila, and right lung compatible with malignancy. The mass results in narrowing of the right hilar structures, the brachiocephalic veins, and superior vena cava.  2. Ill-defined groundglass attenuation in the aerated portion of the right lower lobe which may reflect postobstructive pneumonitis. Subcentimeter nodules may be infectious, inflammatory, or neoplastic in etiology.  3. Trace right pleural effusion and right lower lobe atelectasis.  4. Enlarged paraesophageal and cardiophrenic lymph nodes.  5. Small pericardial effusion.  6. No CT evidence of pulmonary embolus to the segmental level.  CT guided biopsy of the mediastinum:  Nodular sclerosis classic Hodgkin lymphoma  Labs: Unremarkable except for an ESR of 65 | |
| PHYSICAL EXAM FINDINGS | N/A |
| 1. Written in layman’s terms |  |
| 1. General appearance- affect, appearance, position of patient at opening (i.e. sitting, laying down, holding abdomen etc.) | Well appearing female sitting up and in no acute distress but appears anxious and worried. Coughing during encounter. |
| 1. Vital signs | Normal vitals, BP on higher end 135/85 (normal for her) |
| 1. Specific findings and affect |  |
| 1. Response to certain physical movements |  |
|  |  |
|  |  |
| ASSESSMENT | Student will review the findings from the chest X-ray and chest CT and pathology that confirm that you had a mass consistent with lymphoma which is cancer. |
| Additional Discussion | Upon hearing this news, you should appear rather unaffected. Say something like “Okay, well how much time do you think this is going to take to fix it? I just want to feel better so I can go back to my life and make sure I don’t lose my job.”  They should reassure you that your health is the most important thing right now and that we need to keep you safe by getting you started on treatment. They should tell you that we have many resources to help with finances and getting approved time off work.  Also, you should say something like “So does this mean that I can’t try to have a baby now? My husband and I were just talking about that.”  Throughout the process, the student should use good communication skills to discuss this news. They should create an environment and tone that is supportive (e.g. calm voice, prep you for the fact that the test results are not good, and provide empathic reflections on your concerns and worries). |
| PLAN |  |
|  | Student will provide general information about this diagnosis and the type of professionals typically involved in care/treatment. They are not expected to give you a definitive plan. |
| Cancer Workup | Student should go through additional studies that may be involved in your workup and staging including imaging, labs and studies to evaluate your heart and lungs to make sure that you are safe to get therapy. |
| Urgent/Emergent Cancer Treatment | Student should ask about your symptoms of shortness of breath and how severe this is. They should ask if you are having any difficulty swallowing or breathing. They should ask if you are coughing up any blood. |
| Social Factors | Student should ask about your financial concerns and offer resources including referral to social work for financial assistance, etc.  Student should ask about any concerns related to fertility or family planning. |
| Emotional Factors | Student should ask if you would like any resources for emotional support. |
|  |  |

SP Case: Rectal Cancer Patient

Case Adapted from: Cvengros JA, Behel JM, Finley E, Kravitz R, Grichanik M, Dedhia R. Breaking Bad News: A Small-Group Learning Module and Simulated Patient Case for Preclerkship Students. MedEdPORTAL. 2016 Nov 22;12:10505. doi: 10.15766/mep_2374-8265.10505. PMID: 30984847; PMCID: PMC6440412.

Patient Name: Mr./Ms. Terry Allen

Chief Complaint: “I’ve been having blood in my stool, now I’m told I have rectal cancer”

Type and level of learner: first/second year medical student

Case Objectives:

1. Gather an oncology-focused history for a patient with new cancer diagnosis
2. Assess patient needs including urgent/emergent medical care, multidisciplinary referrals, emotional/spiritual needs, interprofessional evaluations

**Suggested Time for Components (Total Encounter Length 20 minutes):**

Introduction: 2 minutes

History of Present Illness: 6 minutes

Review of Systems: 3 minutes

Physical Exam: N/A

Review of imaging and completed workup: 3 minutes

Assessment and Plan: 6 minutes

| SETTING: outpatient, in patient, ED, home, nursing home, rehab, group etc. | outpatient |
| --- | --- |
| PATIENT PROFILE: Information about the “patient” that helps select an SP and helps the learner get an understanding of them as a person. SP will know more information about the patient than learner will ever ask but allows SP to portray a fully developed patient personality. If none of the items below are particulars for the case please write “all may be used.” | |
| Age range | 50-70 (stated age 55) |
| Race and/or ethnic group | All may be used |
| Religious/spiritual background | All may be used |
| Sex (e.g., male, female, intersex, transwoman, transman) | Male |
| Sexual Orientation (e.g., heterosexual, lesbian, gay, bisexual, pansexual, queer, asexual) | Heterosexual |
| Gender expression (e.g., man, woman, gender queer) | All may be used |
| Race/ethnicity: | All may be used |
| Physical description (e.g., BMI, height range) | All may be used |
| Physical limitations (e.g.,) |  |
| Patient appearance (e.g., disheveled, hospital gown, business casual, casual) | Hospital gown, well-groomed. |
| Moulage + location (e.g., none, bruises, scars, body piercing, tattoos) | none |
| Affect (e.g., pleasant, cooperative) | You are appropriately concerned about your diagnosis. You are cooperative with student. |
| Family group (e.g., who is family, who they live with) | Lives with spouse |
| Education | Masters in Business |
| Level of health literacy | Average |
| Employment, if any - present and past, noting any current stresses | Marketing |
| Home/homeless - type of dwelling, number of stories, owned or rented | Rented apartment |
| Financial situation- any current stresses | Recent investment went poorly – causing significant financial strain |
| Insurance Status (e.g., un/under/insured, public/private, HMO/PPO) | Insured |
| Habits (i.e., diet, exercise, caffeine, smoking, alcohol, drugs) | Coffee with breakfast bar in the car on the way to work. Pack lunch most days or get fast food with colleagues. Generally cook at home – combination of prepared and fresh foods. Doesn’t really exercise but tries to get out and do something like walking on the weekends. Smoke ½ pack -1 pack per day since college. Usually drink a beer with dinner, may have 2 on the weekends. |
| Activities (i.e., hobbies, sports, clubs, friends) | Dining out, visiting with friends |
| Typical day - what is the usual daily routine | See Habits, above. Sleeping well, 7-8 hours per night until the last two weeks (since news of possible cancer diagnosis). Now sleeping 5-6 hrs with difficulty falling asleep. |

| CASE INFORMATION | |
| --- | --- |
| Chief Concern: What the patient will say when greeted by the student. The patient’s primary reason for seeking medical care often stated in his/own words. | “My regular doctor referred me here because I’ve been having blood in my stool. I had a colonoscopy and they told me I may have some kind of cancer but I don’t know anything else.” |
| Additional Concerns: Other, if any, concerns the patient has today (i.e., symptoms, requests, expectations, etc.) that will become part of set agenda. |  |
|  | |
| THE PATIENT STORY: The SP will be asked to tell their symptom story and the personal and emotion impact for each of their concerns. You will want to write this is the patient voice. The symptom story should be able to answer this question:“Tell me more about [chief concern/additional concern], starting at the beginning and bringing me up to now.”  The personal context should be able to answer questions concerning the broader personal/psychosocial context of symptoms, especially the patient beliefs/attributions.  The emotional context should be able to ask how are you doing with this, how does this make you feel, how has this affected you emotionally? IMPACT: How has this affected your life? How has this been for your family? | “I’ve been having bright red blood in my stool and occasionally on toilet paper when I wipe. I’ve noticed it on and off for the last year. I didn’t think much of it at first… you know, Dr. Martin recommended I try Preparation-H for possible hemorrhoids (which was really embarrassing) but it didn’t really help and so she referred me to this clinic. It happened 1x a month or so for the first six months, but over the last six months, it has been occurring almost daily with most bowel movements. I’ve lost about 10lbs and find I’m not as hungry as I used to be. Also, my stools are hard and I have pain on the lower left side of my belly when I have a bowel movement. At my last appointment, she gave me the results of the colonoscopy and told me that I may have cancer and need to come here. Lately I find myself worrying about it when I should be focusing on work, and I’ve had some difficulty getting to sleep. |
| HISTORY OF PRESENT ILLNESS: Although some of the HPI will be given in the patient’s symptom story, the learners will expand the story during the direct question section. Below describe the detailed history, usually about the chief concern, which the student must develop in order to make a useful assessment of the problem: | |
|  | |
| Onset (when; gradual or sudden) | First noticed blood in stool about one year ago. |
| Setting (what was going on or where was patient when symptoms first noticed?) |  |
| Duration (how long) | Happened 1x a month or so for the first 6 months. Over the last 6 months, it has been occurring almost daily with most bowel movements |
| Time relationships (frequency, constant or intermittent) |  |
| Location |  |
| Radiation | LLQ pain with BM, nonradiating (if asked) |
| Quality | Bright red blood in stool and occasionally on toilet paper when you wipe. Hard stool with each BM. |
| Amount |  |
| Aggravated by what | Nothing you can identify. |
| Relieved by what | Nothing you can identify. You tried eating more fiber and drinking water but it doesn’t seem to make a difference. You also tried hemorrhoid cream, but it also didn’t make a difference. |
| Associated with what | LLQ pain when having a bowel movement |
| Attitude (what does the patient think is the problem, and how does he/she feel about it) | Since you have been told that you may have cancer, you’ve been worrying more. They worry has been impacting your sleep and your ability to concentrate at work. |
| REVIEW OF SYSTEMS: Significant positives and negatives | |
| 10lb weight loss | No diarrhea |
| Decreased appetite | No rectal pain |
| Hard stool | No epigastric pain |
| No shortness of breath or lightheadedness | No nausea/vomiting |
|  | |
| Past medical history |  |
| Medication allergies (Name and reaction) | None |
| Environmental allergies (Name and reaction) | None |
| Illnesses | None |
| Vaccinations | Up to date |
| Surgeries | none |
| Accidents/ injuries/ trauma | Slipped on the ice hanging Christmas lights one year, sprained your back, no lasting injuries. |
| Hospitalization | none |
|  | |
| Inclusive sexual and reproductive history | |
| Sexual practices  Sexual partners  Protection: Use of safer sex practices  Use of birth control if appropriate  Risk of intimate partner violence | 1, Heterosexual  Condoms  Condoms  None |
| Ob/GYN HISTORY | Age of onset of menses  Age of menopause  Number of pregnancies  Number of live births  Number of miscarriages  Number of abortions |
| Medications | Prescription/dose/reason  Over the counter/dose/reason: Preparation-H (possible hemorrhoids, did not help  Herbs/supplements/dose/reason  Other: |
| Immunizations | X Tetanus  X Flu  X Hepatitis   - Pneumovax - HPV - Other |
| Tobacco products:  X Cigarettes   - Cigar - Pipe - Chew - E-cigarettes | - Never - Past- year started/year quit   X Current   - - Quantity: ½ pack – 1 pack/day   - # of years: ‘since college’ |
| Alcohol  X Beer   - Wine - Liquor - Other | - Never - Past- year started/year quit   X Current   - - Quantity: 1-2 per night   - # of years: 25 years |
| Drugs   - Weed - Cocaine - Heroin - Meth - Other - IV - Inhalants - Other | X Never   - Past- year started/year quit - Current   - Quantity - # of years |
| Diet (describe) | See Habits |
| Exercise (describe) | See Habits |
| List any other important social history or information important to this case | You have never had a colonoscopy because you didn’t want to ‘go through all the prep’. You feel safe at home and in your neighborhood, and have great support from your spouse and extended family (who live in the suburbs) |
| Family history |  |
| Mother, Father, Siblings, Grandparents, and other significant findings. | Mother and father are alive, appropriately aged, and healthy. You have one sister who is two years younger, and two nieces or nephews. One grandparent diet of colon cancer in their 80s. You do not have children. |
|  |  |
| Physical Exam- Deferred  Colonoscopy report:  There is a multilobular polypoid mass located in the proximal rectum extending distally 5cm  Pathology: Biopsies, rectal mass: Invasive moderately differentiated adenocarcinoma, HPV negative  Labs: Unremarkable except for Hemoglobin of 9.5 g/dL | |
| PHYSICAL EXAM FINDINGS | N/A |
| 1. Written in layman’s terms |  |
| 1. General appearance- affect, appearance, position of patient at opening (i.e. sitting, laying down, holding abdomen etc.) |  |
| 1. Vital signs | Normal |
| 1. Specific findings and affect |  |
| 1. Response to certain physical movements |  |
|  |  |
|  |  |
| ASSESSMENT | Student will review the findings from the colonoscopy and pathology that confirm that you had a mass consistent with rectal cancer. |
| Additional Discussion | Upon hearing this news, you should appear shocked and disbelieving the cancer diagnosis. “How can this be happening to me?”  Become appropriately distressed. Take several moments and “collect yourself” and say, “I was hoping there was a chance that it wasn’t really cancer. This is not what I was expecting, but what do we do from here?”  Throughout the process, the student should use good communication skills to discuss this news. They should create an environment and tone that is supportive (e.g. calm voice, prep you for the fact that the test results are not good, and provide empathic reflections on your concerns and worries).  Phrases such as “this is not what I was expecting” and “how am I am going to tell Caitlin” and “I knew I should have said something to my doctor sooner” and questions like “did I do something wrong?” are all good expressions of your fear and distress.  Also make a comment about financial strain, “how will I pay for this?” |
| PLAN |  |
|  | Student will provide general information about this diagnosis and the type of professionals typically involved in care/treatment. They are not expected to give you a definitive plan. |
| Cancer Workup | Student should go through additional studies that may be involved in your workup and staging including imaging, additional endoscopic procedures like repeat colonoscopy/proctoscopy, etc. |
| Urgent/Emergent Cancer Treatment | Student should review low hemoglobin values and caution regarding symptoms of acute anemia and need for urgent evaluation. Student should also review modifications to help with constipation and caution regarding need for evaluation if unable to pass bowel movements. |
| Social Factors | Student should ask about your financial concerns and offer resources including referral to social work for financial assistance, etc. |
| Emotional Factors | Student should ask if you would like any resources for emotional support. |
