# Supplementary material for: Approach to an Initial Oncologic Patient Encounter: A Simulation-Based Training for First-Year Medical Students
Source: MedEdPORTAL. 2026 Apr 24;22:11574. doi: 10.15766/mep_2374-8265.11574 (PMC13106612; doi:10.15766/mep_2374-8265.11574)
Supplement: Supplementary file 1 — Approach to an Initial Oncologic Patient Encounter.pptxCase Guide for Students.docxCase Information.docxDebrief Guide for Sim Facilitator.docxPostsimulation Evaluation (Original).docxPostsimulation Evaluation (Revised).docx [file mep_2374-8265.11574-s001.zip › D. Debrief Guide for Sim Facilitator.docx]

**Debrief Guide for Simulation Facilitator**

**Instructions for Use:** This guide provides a structured framework for the post-simulation reflection session. It ensures that all learning objectives are met within the time constraints. Follow the numbered sections 1–10, moving from a recap of the medical facts to a discussion on communication effectiveness and clinical reasoning.

This debrief is a collaborative group process, not a lecture. It is not the faculty's responsibility to provide all the feedback. Use the prompts below to facilitate student self-reflection and peer-to-peer feedback before offering your own insights.

As noted in the guide, you may not be able to cover every point due to time constraints; highlight the most pertinent sections based on the students' actual performance.

**Debrief Guide for Simulation Facilitator: Oncology Focused History**

1. Recap the Simulation Scenario:

• Briefly summarize the case presented to the students. Highlight key details such as patient demographics, presenting symptoms, and relevant medical history.

2. Discuss Performance Objectives:

• Review the specific objectives of the simulation. This may include:

o Gathering a comprehensive oncology-focused medical history.

o Discussing the components of a physical examination relevant to oncology. (Note: Explain that while a focused physical exam is critical in real-life practice, for the purposes of this simulation, it may be verbalized or abbreviated.

o Demonstrating effective communication skills with the patient.

3. Evaluate History Taking:

• Discuss how well students elicited and documented pertinent information related to oncology:

o Did they ask open-ended questions to explore symptoms?

o Were they able to obtain a detailed past medical history including any previous oncological treatments or family history of cancer?

o Assess the accuracy and completeness of the information gathered.

4. Review Communication Effectiveness:

• Analyze how well students communicated with the standardized patient:

o Were they empathetic and respectful?

o Did they explain procedures and findings clearly?

o Evaluate their ability to manage difficult conversations, such as discussing potential cancer diagnoses or treatment options.

5. Provide Constructive Feedback:

• Student Self-Reflection: Before offering external feedback, ask the student to evaluate their own performance.

o Prompts: "How do you think that went?" "What went well?" "What is one thing you would do differently next time?"

• Student-Directed Focus: Ask the student to identify areas where they specifically want feedback.

o Prompts: "Was there a specific moment where you felt stuck?" "Is there a specific skill you are trying to improve that you want us to comment on?"

• Facilitate Peer Feedback: Ask the student if they would like to hear what their peer observers thought went well first, followed by suggestions.

o Prompts to group: "What did you observe during your peer's interaction?" "Let's start with what was effective." "What constructive suggestions do you have?" Ensure a supportive atmosphere.

• Faculty Feedback: Offer specific feedback on strengths and areas for improvement, filling in gaps not covered by the group.

o Highlight instances where students excelled in history taking, physical examination verbalization, or communication.

o Address any gaps in knowledge, skills, or approach observed during the simulation.

o Encourage reflective practice and self-assessment.

6. Discuss Clinical Reasoning:

• Explore the students' clinical reasoning process:

o Did they formulate an appropriate plan of care based on the history and patient workup?

o How well did they prioritize investigations or referrals?

7. Facilitate Peer Feedback:

• Encourage students to provide feedback to each other:

o Discuss what they observed during their peers' interactions with the patient.

o Encourage a supportive and constructive atmosphere for learning from each other's experiences.

8. Reinforce Learning Objectives:

• Summarize key learning points from the simulation:

o Emphasize the importance of a thorough oncology-focused history *and physical* in clinical practice.

o Discuss strategies for continuous improvement in patient care skills.

9. Encourage Reflection and Action Planning:

• Prompt students to reflect on their performance:

o Ask them to identify specific areas they plan to work on to enhance their oncology patient assessment skills.

o Discuss resources or further training opportunities available to support their learning.

10. Conclude with Q&A and Closure:

• Allow time for students to ask questions or seek clarification on any aspects of the simulation.

• Provide final thoughts and summarize the overall objectives and outcomes of the session.

By following this debrief guide, facilitators can effectively reinforce learning objectives, provide targeted feedback, and encourage continuous improvement in oncology-focused patient assessment skills among medical students. Facilitators should highlight the most pertinent sections from the guide above. It may not be possible to thoroughly discuss each point given time constraints.
