# Supplementary material for: Approach to an Initial Oncologic Patient Encounter: A Simulation-Based Training for First-Year Medical Students
Source: MedEdPORTAL. 2026 Apr 24;22:11574. doi: 10.15766/mep_2374-8265.11574 (PMC13106612; doi:10.15766/mep_2374-8265.11574)
Supplement: Supplementary file 1 — Approach to an Initial Oncologic Patient Encounter.pptxCase Guide for Students.docxCase Information.docxDebrief Guide for Sim Facilitator.docxPostsimulation Evaluation (Original).docxPostsimulation Evaluation (Revised).docx [file mep_2374-8265.11574-s001.zip › E. Postsimulation Evaluation (Original).docx]

**Post Simulation Evaluation (Original)**

**Instructions for Use:** Distribute this form to collect quantitative Likert-scale data and qualitative free-response feedback regarding the students' preparation, confidence, and satisfaction with the simulation.

Please select the answer that best describes your experience. Please complete the free response questions by writing in your answer.

During the simulation, I was a: participant observer

I was given adequate preparation and education prior to the simulation experience.

Strongly Agree Agree Neutral Disagree Strongly Disagree

I was well oriented to the purpose of the simulation.

Strongly Agree Agree Neutral Disagree Strongly Disagree

I was adequately oriented to the clinical simulation environment.

Strongly Agree Agree Neutral Disagree Strongly Disagree

The simulation helped me to apply my knowledge to a practical application.

Strongly Agree Agree Neutral Disagree Strongly Disagree

Clinical simulation is a valuable tool that enables me to practice my clinical skills.

Strongly Agree Agree Neutral Disagree Strongly Disagree

I felt comfortable in sharing my thoughts and feelings during the group debriefing session.

Strongly Agree Agree Neutral Disagree Strongly Disagree

The debriefing session helped me to critically reflect upon the performance of the group.

Strongly Agree Agree Neutral Disagree Strongly Disagree

I plan to use what I learned today in simulation in my future clinical practice.

Strongly Agree Agree Neutral Disagree Strongly Disagree

1. Identify 3 concepts that you learned as an observer of the simulation.
2. Identify 3 concepts that you learned as a participant of the simulation (for participants only).
3. What aspects of the simulation were least helpful to you?
4. What aspects of the simulation were most helpful to you?
5. What would you do differently as a participant of the simulation (for participants only)?
6. Please provide any suggestions that might improve future simulation experiences.
7. Do you feel the facilitator(s) of the debriefing session was helpful to your learning experience?

Please provide any additional feedback.
