# Supplementary material for: Approach to an Initial Oncologic Patient Encounter: A Simulation-Based Training for First-Year Medical Students
Source: MedEdPORTAL. 2026 Apr 24;22:11574. doi: 10.15766/mep_2374-8265.11574 (PMC13106612; doi:10.15766/mep_2374-8265.11574)
Supplement: Supplementary file 1 — Approach to an Initial Oncologic Patient Encounter.pptxCase Guide for Students.docxCase Information.docxDebrief Guide for Sim Facilitator.docxPostsimulation Evaluation (Original).docxPostsimulation Evaluation (Revised).docx [file mep_2374-8265.11574-s001.zip › F. Postsimulation Evaluation (Revised).docx]

**Post Simulation Evaluation (Revised)**

**Instructions for Use:** Distribute this form to collect quantitative Likert-scale data and qualitative free-response feedback regarding the students' preparation, confidence, and satisfaction with the simulation.

Please select the answer that best describes your experience. Please complete the free response questions by writing in your answer.

During the simulation, I was a: participant observer

How adequate was the preparation and education you received prior to the simulation experience?

(1) Extremely adequate

(2) Quite adequate

(3) Moderately adequate

(4) Slightly adequate

(5) Not at all adequate

How well were you oriented to the purpose of the simulation?

(1) Extremely well

(2) Quite well

(3) Moderately well

(4) Slightly well

(5) Not at all well

How well were you oriented to the clinical simulation environment?

(1) Extremely well

(2) Quite well

(3) Moderately well

(4) Slightly well

(5) Not at all well

How helpful was the simulation in applying your knowledge to a practical application?

(1) Extremely helpful

(2) Quite helpful

(3) Moderately helpful

(4) Slightly helpful

(5) Not at all helpful

How valuable was the simulation for practicing your clinical skills?

(1) Extremely valuable

(2) Quite valuable

(3) Moderately valuable

(4) Slightly valuable

(5) Not at all valuable

How comfortable did you feel sharing your thoughts and feelings during the group debriefing session?

(1) Extremely comfortable

(2) Quite comfortable

(3) Moderately comfortable

(4) Slightly comfortable

(5) Not at all comfortable

How effective was the debriefing session in helping you critically reflect upon the group's performance?

(1) Extremely effective

(2) Quite effective

(3) Moderately effective

(4) Slightly effective

(5) Not at all effective

How helpful was the facilitator(s) of the debriefing session to your learning experience?

(1) Extremely helpful

(2) Quite helpful

(3) Moderately helpful

(4) Slightly helpful

(5) Not at all helpful

How likely are you to use what you learned in the simulation today in your future clinical practice?

(1) Extremely likely

(2) Quite likely

(3) Moderately likely

(4) Slightly likely

(5) Not at all likely

1. Identify 3 concepts that you learned as an observer of the simulation.
2. Identify 3 concepts that you learned as a participant of the simulation (for participants only).
3. What aspects of the simulation were least helpful to you?
4. What aspects of the simulation were most helpful to you?
5. What would you do differently as a participant of the simulation (for participants only)?
6. Please provide any suggestions that might improve future simulation experiences.
7. Do you feel the facilitator(s) of the debriefing session was helpful to your learning experience?

Please provide any additional feedback.
